# Supplementary material for: Intestinal NUCB2/nesfatin-1 regulates hepatic glucose production via the MC4R-cAMP-GLP-1 pathway
Source: EMBO J. 2024 Nov 19;44(1):54–74. doi: 10.1038/s44318-024-00300-4 (PMC11696497; doi:10.1038/s44318-024-00300-4)
Supplement: Supplementary file 1 — Appendix [file 44318_2024_300_MOESM1_ESM.pdf]

APPENDIX

Intestinal NUCB2/nesfatin-1 regulates hepatic glucose production *via* the MC4R-cAMP-GLP-1 pathway

Shan Geng *et al.*

Lead contact: mengliu.yang@cqmu.edu.cn

This file includes:

Appendix Figures S1 to S9
Appendix Tables S1 to S3

Appendix Figures

A

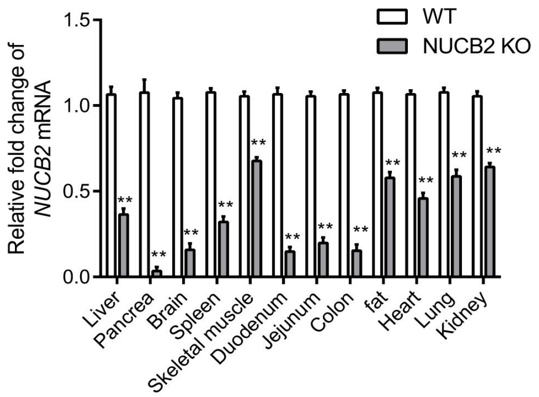

B

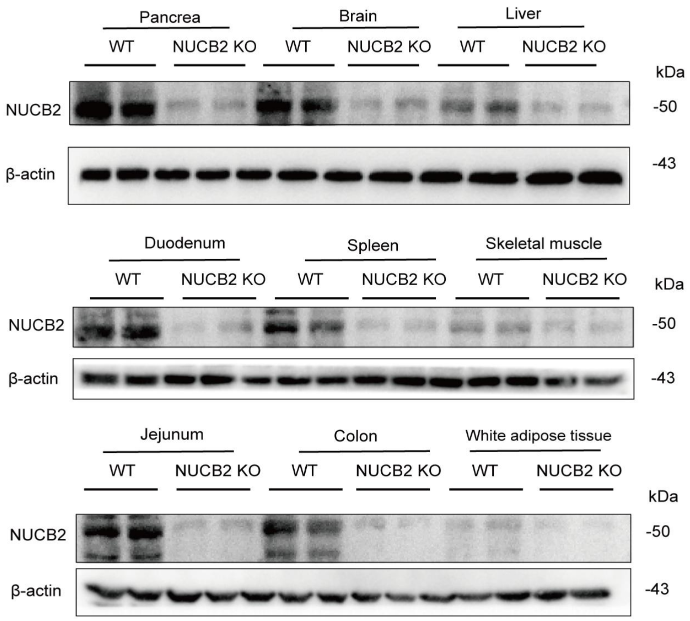

Appendix Figure S1. NUCB2 expression in different tissues of WT and NUCB2-KO rats. **(A)** NUCB2 mRNA expression in different tissues. **(B)** NUCB2 protein expressions in different tissues. Values were shown as mean ± SEM (n = 3 rats for each group). Unpaired Student's *t*-test was used for statistical analysis. $^{**}p < 0.01$ *vs.* WT rats.

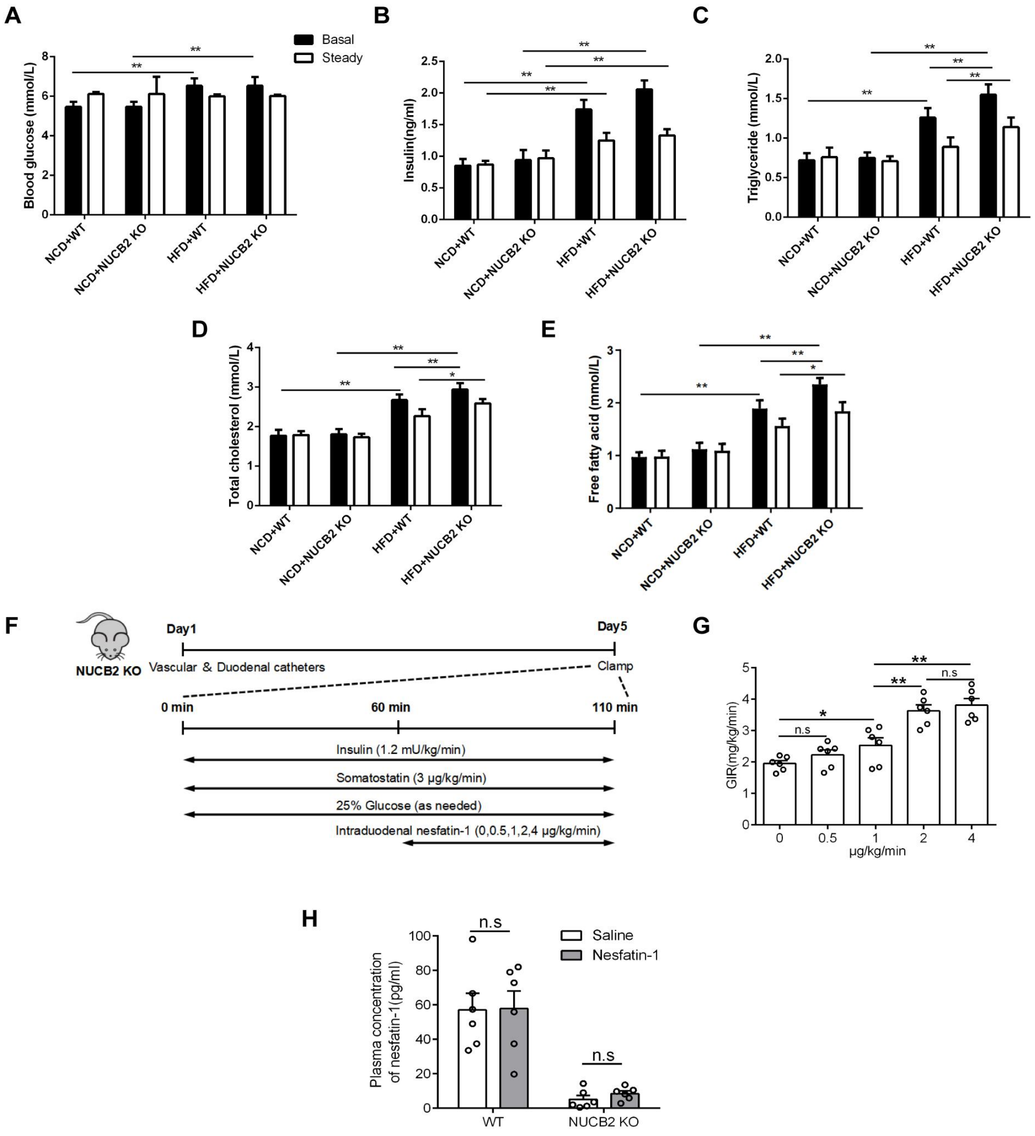

Appendix Figure S2. Serum biochemistry parameters in intestinal glucose infusion and effect of intestinal nesfatin-1 infusion on GIR during the PEC. (A-E) Blood glucose (A), insulin (B) triglyceride (C), total cholesterol (D) and free fatty acid (E) in WT and NUCB2 KO rats during the PEC under intestinal glucose infusion. **(F)** Schematic representation of the experimental design. NUCB2 KO rats received different concentrations of nesfatin-1 infusion in the duodenum and a PEC experiment was performed. **(G)** GIR during the clamp. **(H)** circulating nesfatin-1 concentration after duodenal nesfatin-1 infusion. NCD, normal chow diet; HFD, high-fat diet; GIR, glucose infusion rate; n.s, not significant. Values were shown as mean ± SEM (n = 6 rats). Two-way ANOVA followed by Bonferroni's test was used for **(A-E**

and H) and one-way ANOVA followed by Bonferroni's test was used for **(G)**. *$p < 0.05$**,** **$p < 0.01$.

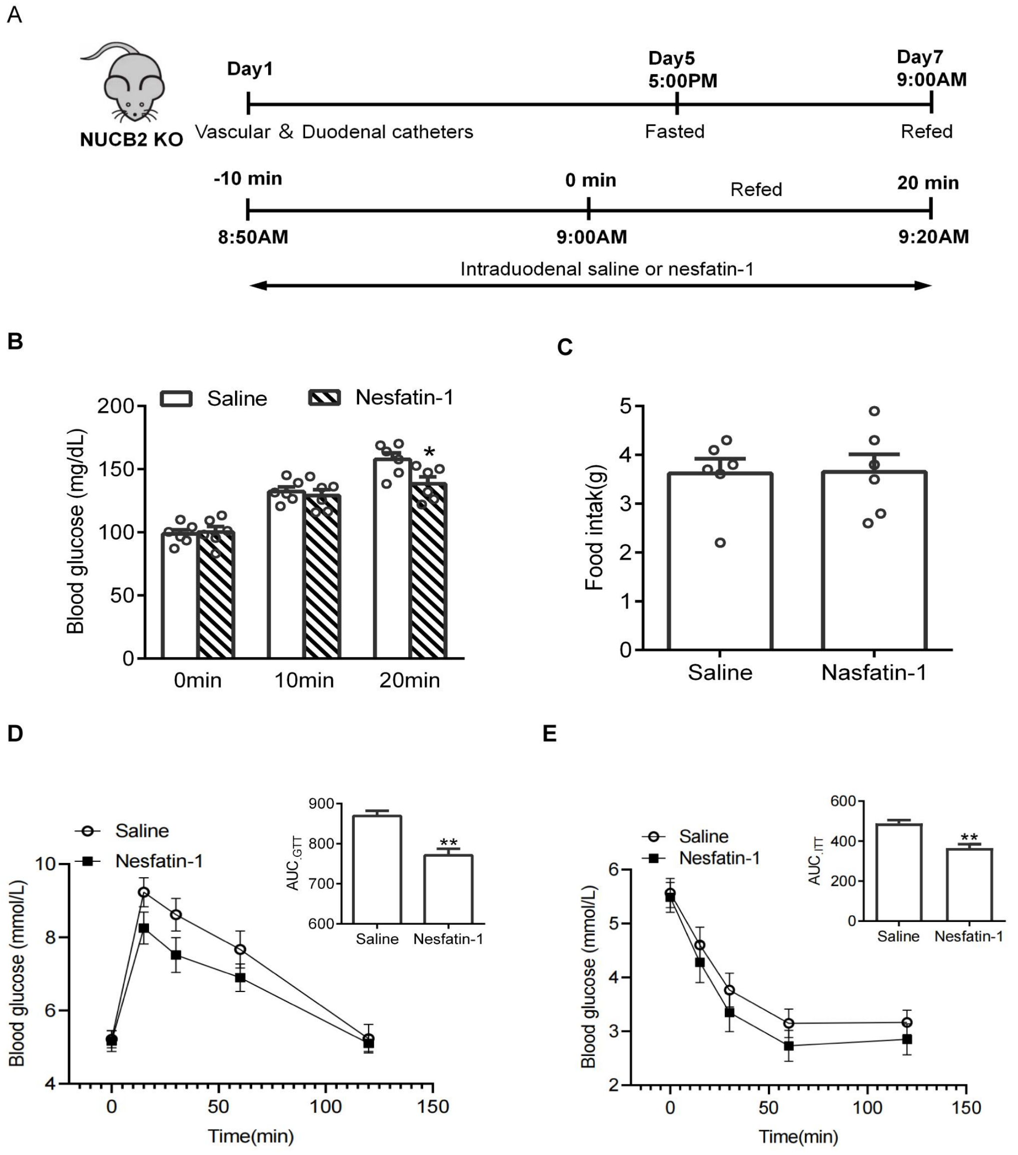

Appendix Figure S3. Effect of gut nesfatin-1 on blood glucose *in vivo*. (A-C) Schematic representation of the experimental design for a fasting-refeeding experiment (A). Blood glucose levels (B) Food intake during feeding (C). **(D and E)** 8-week-old male NUCB2-KO rats were fed an NCD for 12 weeks. Nesfatin-1 or saline was infused through a duodenal catheter during the GTT or ITT. Blood glucose and AUC during the GTT (D). Blood glucose and AUC during the ITT (E). NCD, normal chow diet; GTT, glucose tolerance tests; ITT, insulin tolerance tests. AUC, areas under the curves. Values were shown as mean ± SEM (n = 6 rats). Two-way ANOVA followed by Bonferroni's test was used for (B) and unpaired Student's *t*-test was used for (C-E). *$p < 0.05$, **$p < 0.01$ *vs.* saline.

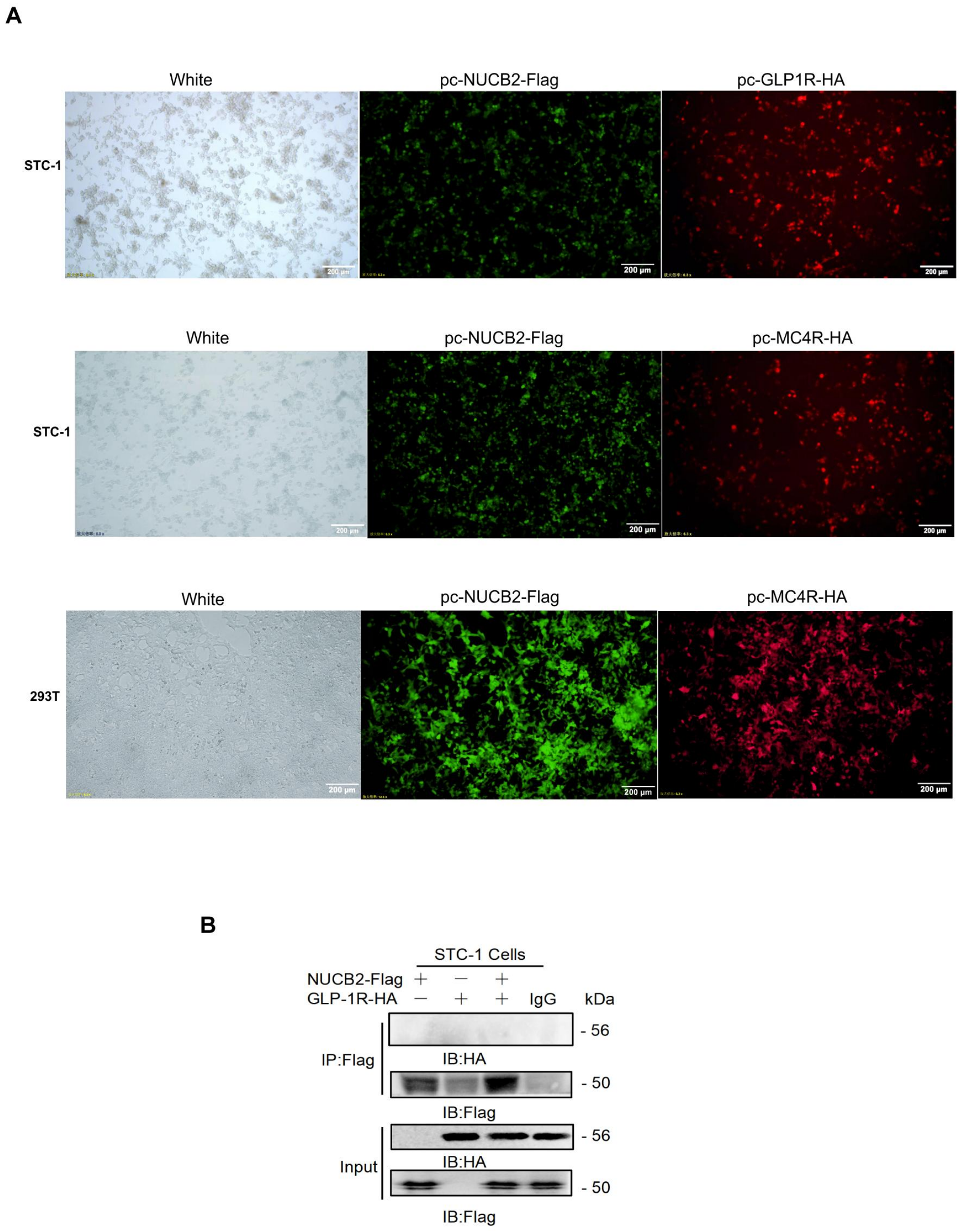

Appendix Figure S4. NUCB2, GLP-1R, and MC4R expression and Co-IP experiment for NUCB2 and GLP-1RA *in vitro.* (A) STC-1 or 293T cells were transfected with pc*NUCB2-Flag* and pc*MC4R*-HA or pc*GLP-1R*-HA, respectively. IF staining for NUCB2 and GLP-1R expression or NUCB2 and MC4R expression in STC-1 cells (upper and middle

panels) and in 293T cells (bottom panel). **(B)** STC-1 cells were transfected with NUCB2-Flag or/and GLP-1R-HA. Co-IP was performed using an anti-HA antibody (n = 3 independent experiments).

A

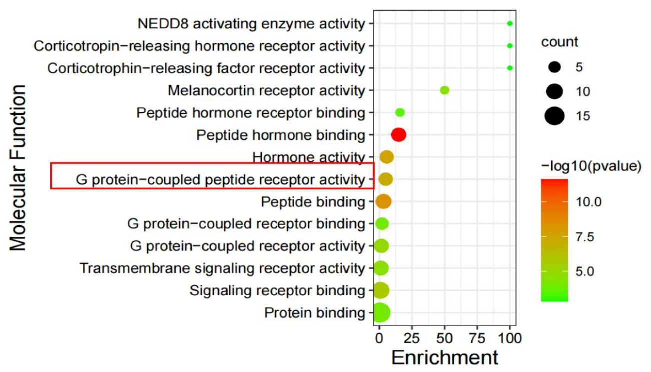

B

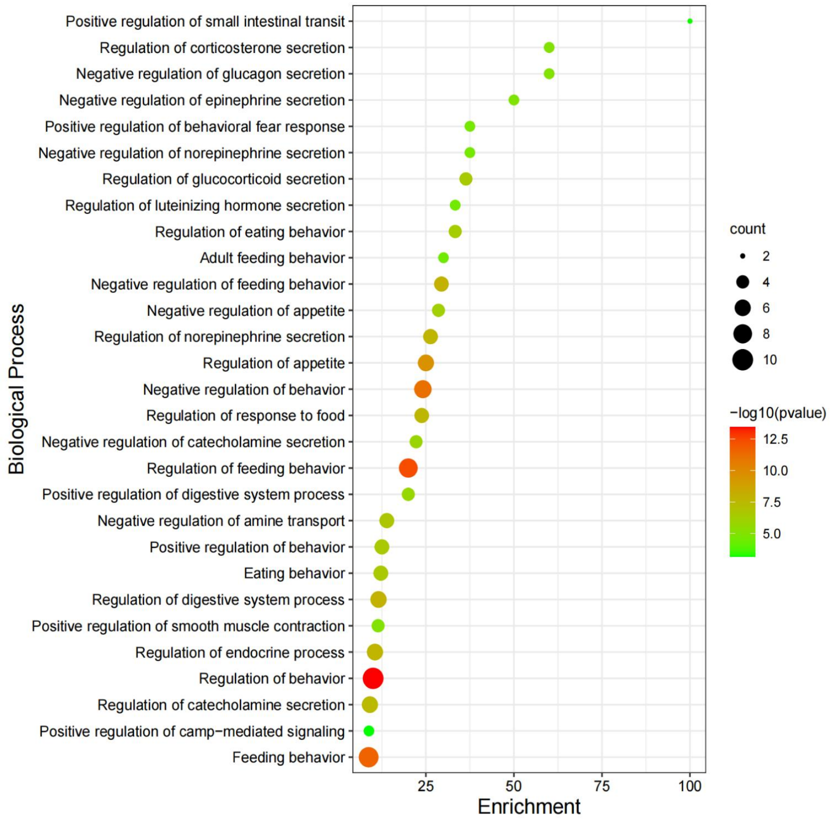

Appendix Figure S5. GO analysis of DEGs. (A) GO analysis of DEGs for MF. **(B)** GO analysis of DEGs for BP. GO, Ontology analysis; DEG, Differentially expressed gene; MF, Molecular Function; BP, Biological process.

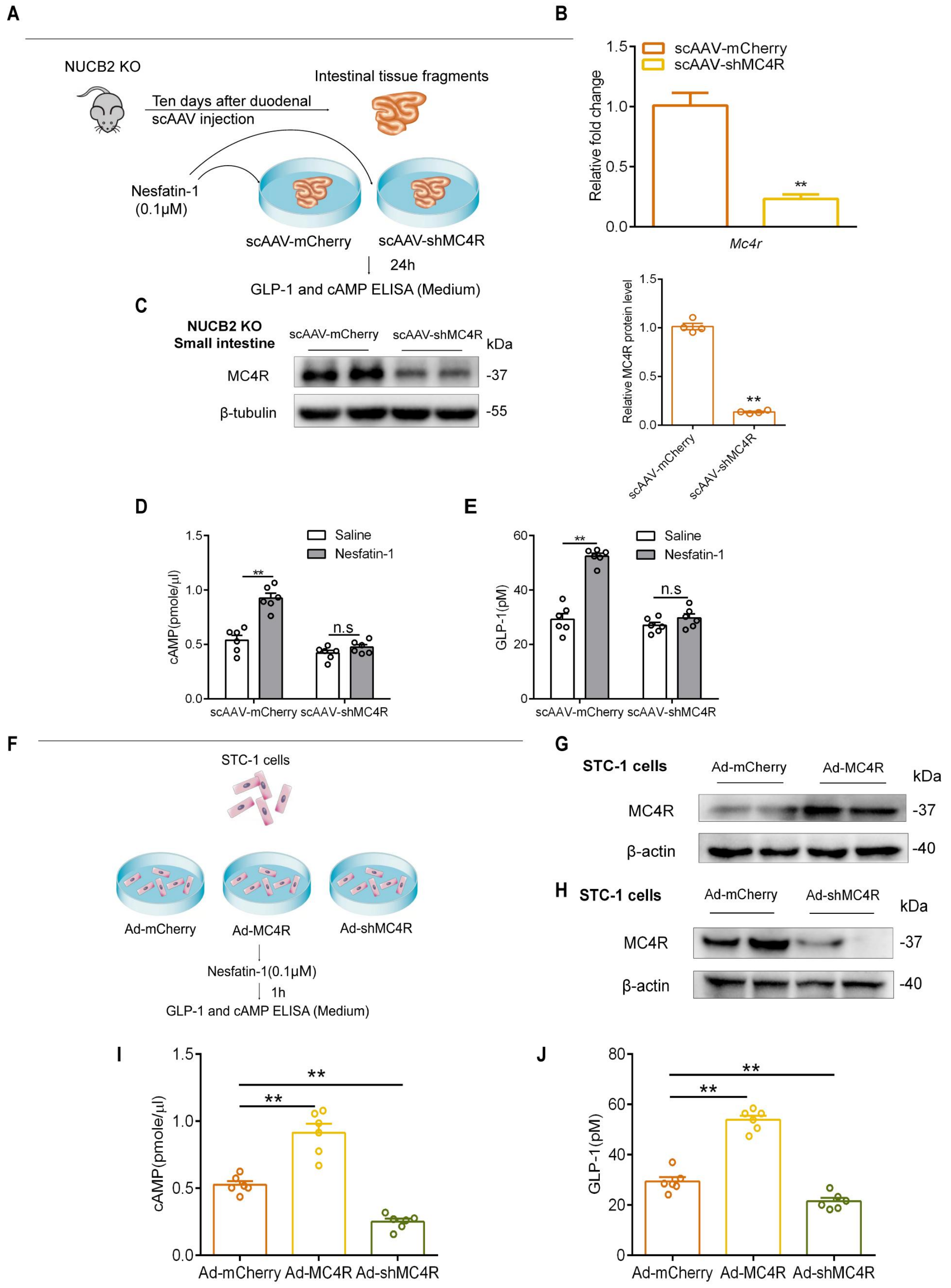

Appendix Figure S6. Association of nesfatin-1 with the MC4R-cAMP signaling pathway *in vitro*. NUCB2 KO mice were duodenally infected with scAAV-shMC4R or scAAV-mCherry. Ten days post-infection, the small intestinal fragments were then sectioned, cultured and treated with nesfatin-1 for 24 h. **(A)** Experimental procedure. **(B)** MC4R mRNA expression and **(C)** protein expression. cAMP **(D)** and GLP-1 **(E)** levels in the culture medium of small intestinal fragments were determined by ELISA. **(F)** STC-1 cells treated

with Ad-MC4R, Ad-shMC4R or Ad-mCherry, and then cultured with nesfatin-1 for 1 h. **(G and H)** MC4R protein expression of STC-1 cells treated with Ad-MC4R (G) and Ad-shMC4R (H). The cAMP (**I**) and GLP-1 **(J)** concentration in the culture medium of STC-1 cells was determined by ELISA. n.s, not significant. Values were shown as mean ± SEM (n = 4 - 6 rats or 4 - 6 independent experiments). Unpaired Student's *t*-test was used for **(B, C)**, Two-way ANOVA followed by Bonferroni's test was used for **(D, E)**, and one-way ANOVA followed by Bonferroni's test was used for **(I, J)**. $^{**}p < 0.01$.

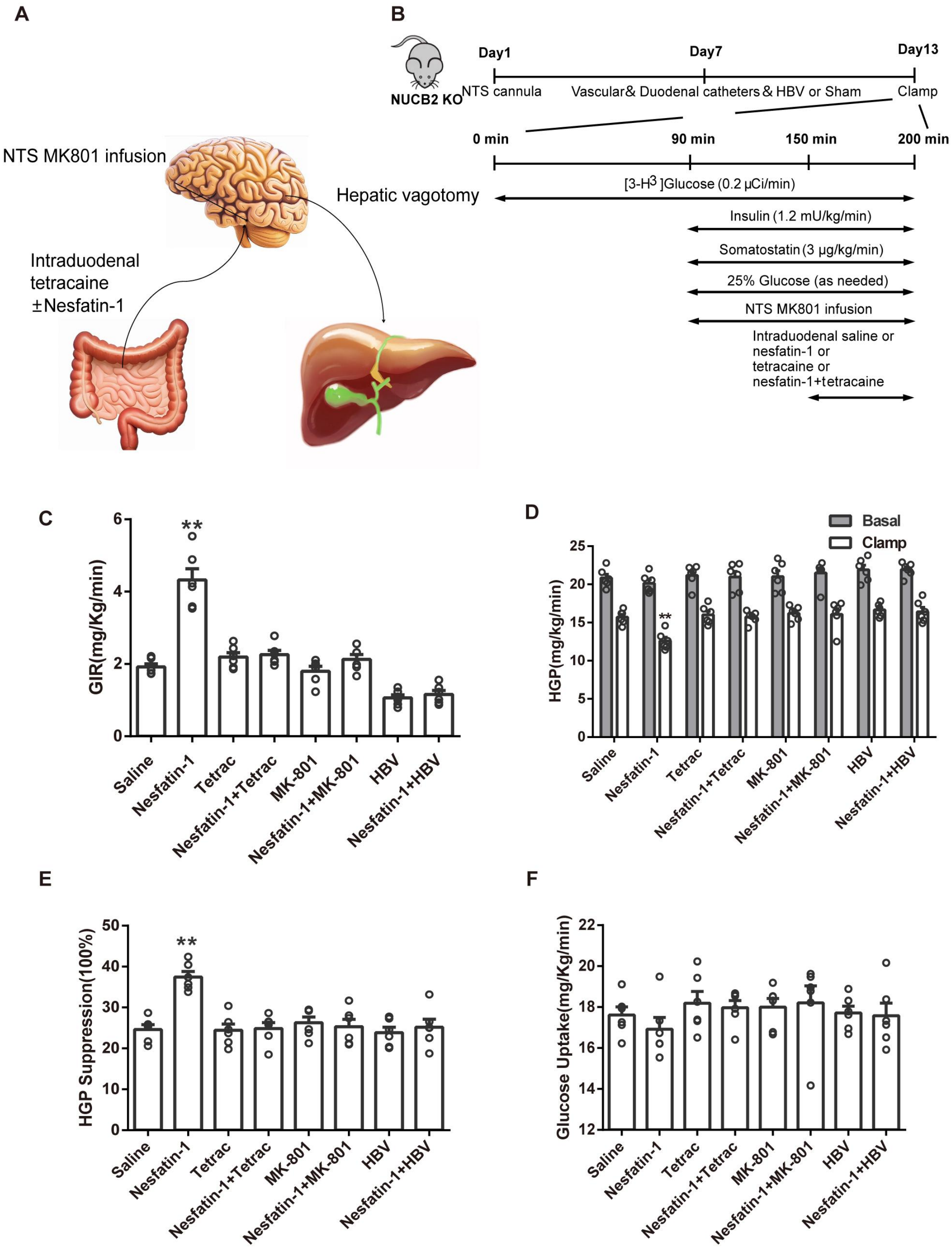

Appendix Figure S7. Gut nesfatin-1 inhibits hepatic glucose production through a gut-brain-liver neurocircuitry. (A) Experimental design. **(B)** Experimental procedure and clamp protocol. **(C)** GIR. **(D)** HGP. **(E)** HGP suppression. **(F)** Glucose uptake. HBV, hepatic branch vagotomy; GIR, glucose infusion rate; HGP, hepatic glucose production. Values are shown as mean ± SEM (n = 6 rats). One-way ANOVA followed by Bonferroni's test was used for statistical analysis. $^{*}p < 0.05$, $^{**}p < 0.01$ *vs*. other groups.

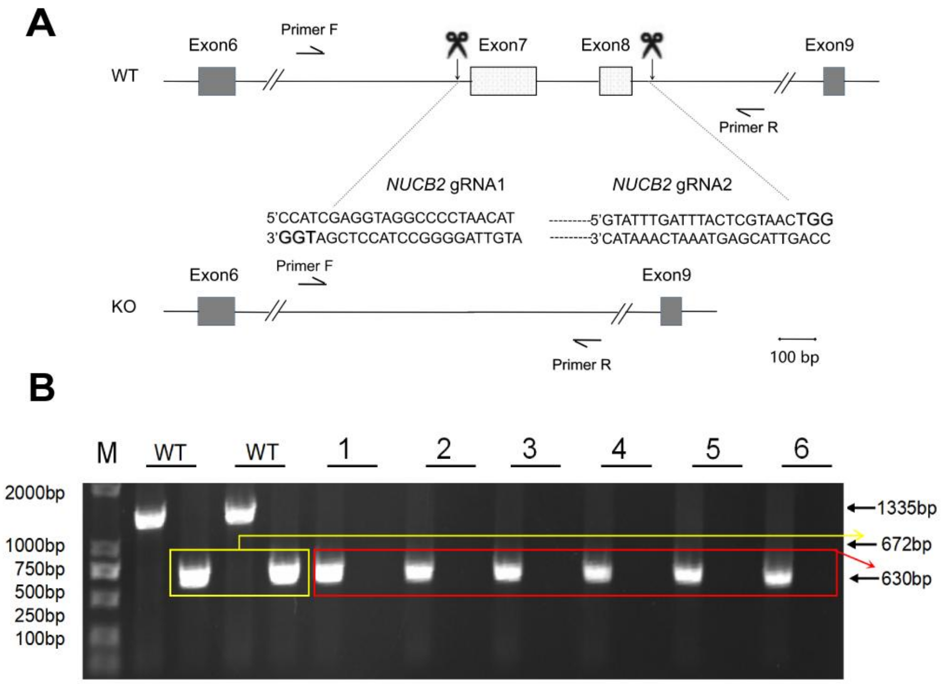

Appendix Figure S8. Identification of genotype in rats. (A) Schematic representation of the paired-KO strategy for NUCB2 knockout. Primer F and Primer R are primers used for PCR amplification. **(B)** Genomic DNA PCR results. M: DNA ladder 2000; WT, wild-type rat; 1-6, NUCB2-KO rats.

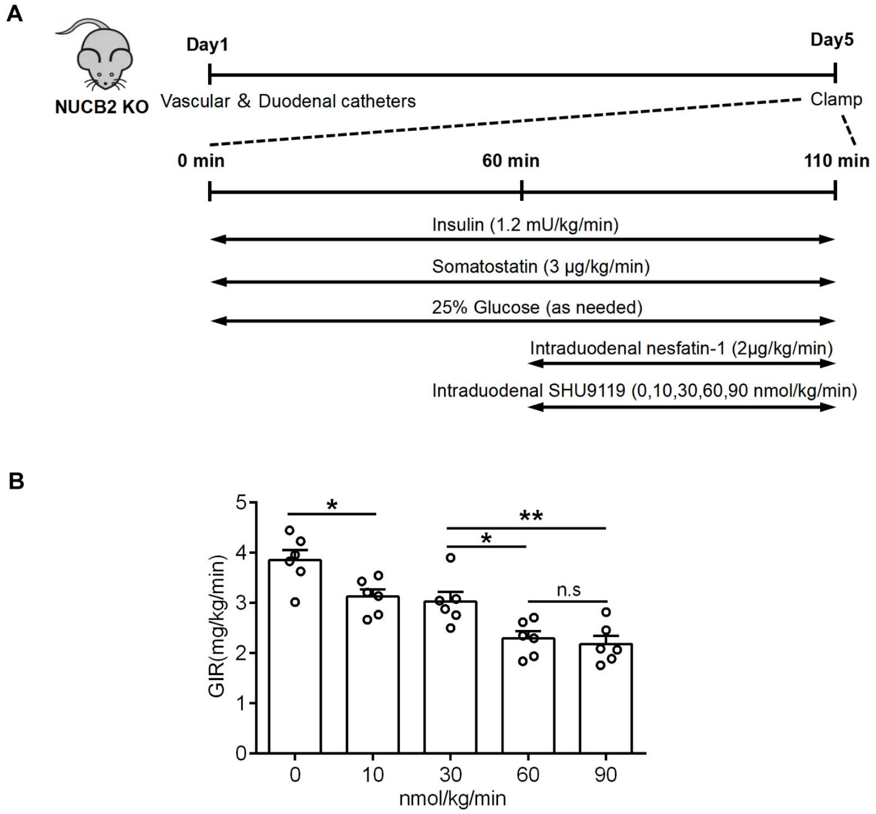

Appendix Figure S9. Effect of intestinal SHU 9119 infusion on GIR during the PEC. (A) Schematic representation of an experimental design. NUCB2 KO rats received duodenal nesfatin-1 (2μg/kg/min) and different concentrations of SHU9119 infusion, followed by a PEC experiment. **(B)** GIR during the clamp. n.s, no significance. PEC, pancreatic-euglycemic clamp; GIR, glucose infusion rates. Values are shown as mean ± SEM (n = 6 rats). One-way ANOVA followed by Bonferroni's test was used for statistical analysis. *$P < 0.05$, **$P < 0.01$.

Appendix Tables

Appendix Table S1. Primer sequences for quantitative RT-PCR

Gene	Sequence (5'-3')
Pck1	Forward: TGCCAGCCAGAGTATATTC<br>Reverse: TGAGAGCCAGCCAACA
G6pc1	Forward: TGGTGGCTGGAGTCTTG<br>Reverse: TCTGGAGGCTGGCATTG
Mc4r	Forward: ATCTGTAGCTCCTTGCTCGC<br>Reverse: TGCAAGCTGCCCAGATACAA
β-actin	Forward: CCATTGAACACGGCATTG<br>Reverse: TACGACCAGAGGCATACA

Appendix Table S2. Plasmid sequence designed for co-IP experiment

Name	Sequence (5’-3’)
pc-NUCB2-Flag	Forward: CTTGGTACCGAGCTCGGATCCGCCACCATGAGGTGGAGGACCATCCTGC<br>Reverse: GAAGGGCCCTCTAGACTCGAGAATGTGTGGCTCAAACTTCAATTCTCC
pc-MC4R-HA	Forward: GAGGATCCCCGGGTACCGGTCGCCACCATGAACTCCACCCACCACCATG<br>Reverse: CACACATTCCACAGGCTAGCTTAGGCGTAGTCAGGCACGTCATAAGGGTAAGCATAG
pc-GLP-1R-HA	Forward: CTTGGTACCGAGCTCGGATCCGCCACCATGGCCAGCACCCCAAGC<br>Reverse: GAAGGGCCCTCTAGACTCGAGGCTGTAGGAACTCTGGCA
pEGFP-NUCB2	Forward:CTACCGGACTCAGATCTCGAGCCACCATGAGGTGGAGGATCATC<br>REVERSE:GTACCGTCGACTGCAGAATTCGTGTGTGTGGCTCAAACTT
pmCherry-MC4R	Forward: CTACCGGACTCAGATCTCGAG<br>Reverse:GTACCGTCGACTGCAGAATTC

Appendix Table S3. Plasmid sequence designed for mutagenesis

Name	Sequence (5'-3')
pc-ΔEF-Flag (NUCB2 247aa-322aa mutant)	Forward: TTGACCCCGAAAAAAAAGAATTCTTGGAGCCAGATAGCTG<br>Reverse: TTCTTTTTTTTCGGGGTCAAAGTCATTAGGATCCAATCCAT
pc-ΔDNA- Flag (NUCB2-171aa-223aa mutant)	Forward: ACACTATGACGGAAGCAAAGATCAACTAAAAGAGGTATGGG<br>Reverse: CTTTGCTTCCGTCATAGTGTTCCAGATCACTTGTTGCC
pc-Δ25-50-Flag (NUCB2-25aa-50aa mutant)	Forward: TCTTGAAGCTGGACTTTATTATGATGAATATCTCAAGCAAGTGATTG<br>Reverse: AATAAAGTCCAGCTTCAAGAGCAGTAAGTAAACATGTAATCAAG
pc-Δ51-75-Flag (NUCB2-51aa-75aa mutant)	Forward: CACCAGATACTCAGAAAGCAGACATAGAGGAAATAAAGAGTGG<br>Reverse: TGCTTTCTGAGTATCTGGTGGTTCTATCTTCGCACT
pc-Δ76-106-Flag (NUCB2-76aa-106aa mutant)	Forward: AGAAAAGCTCAAAAGGCAAGAAGTAGGAAGGTTAAGAATGT<br>Reverse: CTTGCCTTTTGAGCTTTTCTCTGAAGTGTTTATCTGTTTCC
pc-NUCB2$^{\Delta HFR}$-Flag (NUCB2-$_{70}$HFR$_{72}$ mutant)	Forward: CACACTGGACTAGTGGATCCCGCCACCATGAGGTGGAGGACCATCCAAG<br>Reverse: AGTCACTTAAGCTTGGTACTGTGTGTGGCTCAAACTTCAGCTC
