# Supplementary figures and images for: Intestinal NUCB2/nesfatin-1 regulates hepatic glucose production via the MC4R-cAMP-GLP-1 pathway
Source: EMBO J. 2024 Nov 19;44(1):54–74. doi: 10.1038/s44318-024-00300-4 (PMC11696497; doi:10.1038/s44318-024-00300-4)

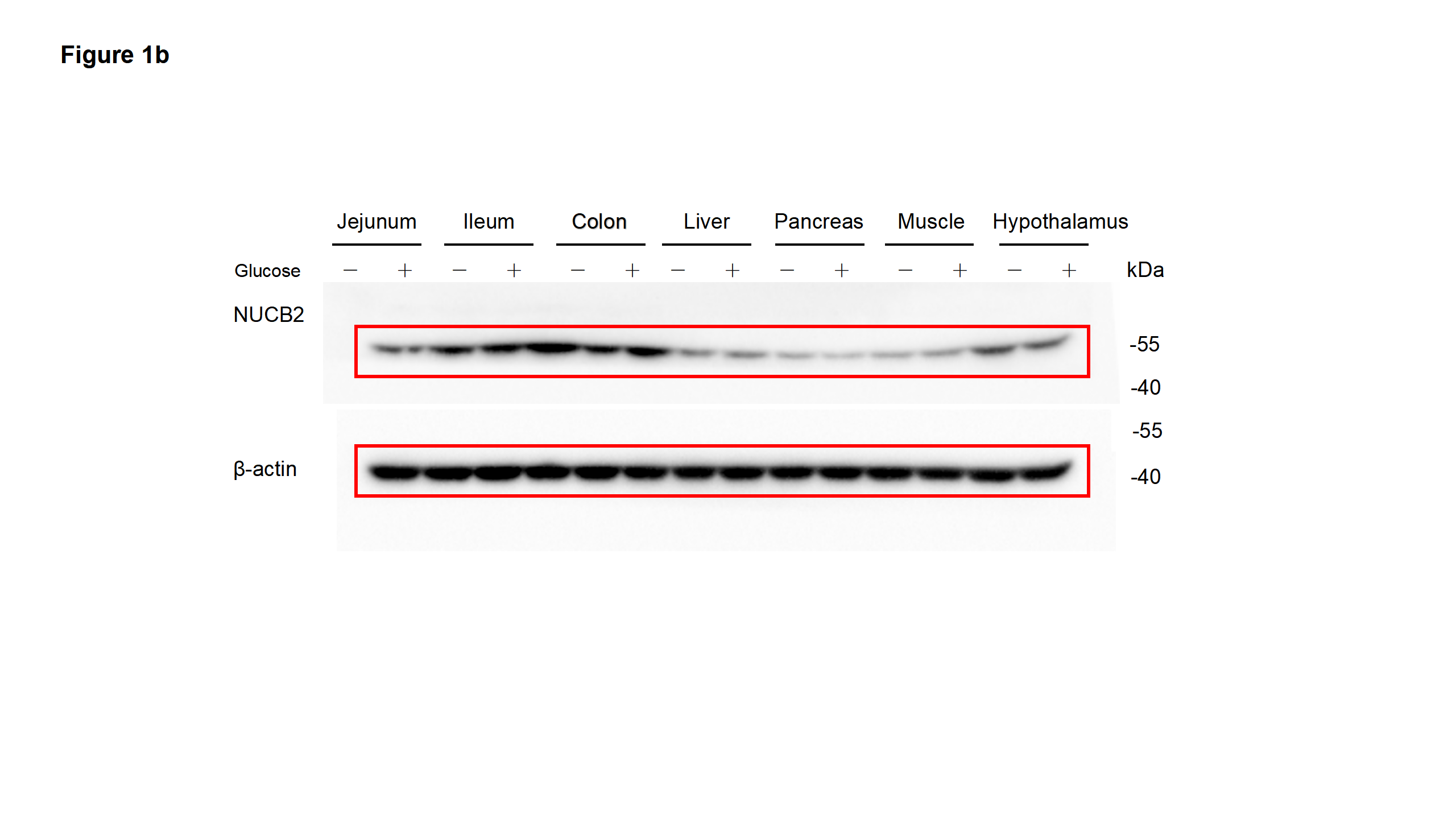

Supplement: Supplementary file 3 — Source data Fig. 1 [file 44318_2024_300_MOESM3_ESM.zip › Figure 1/fig1b/fig1b.tif]

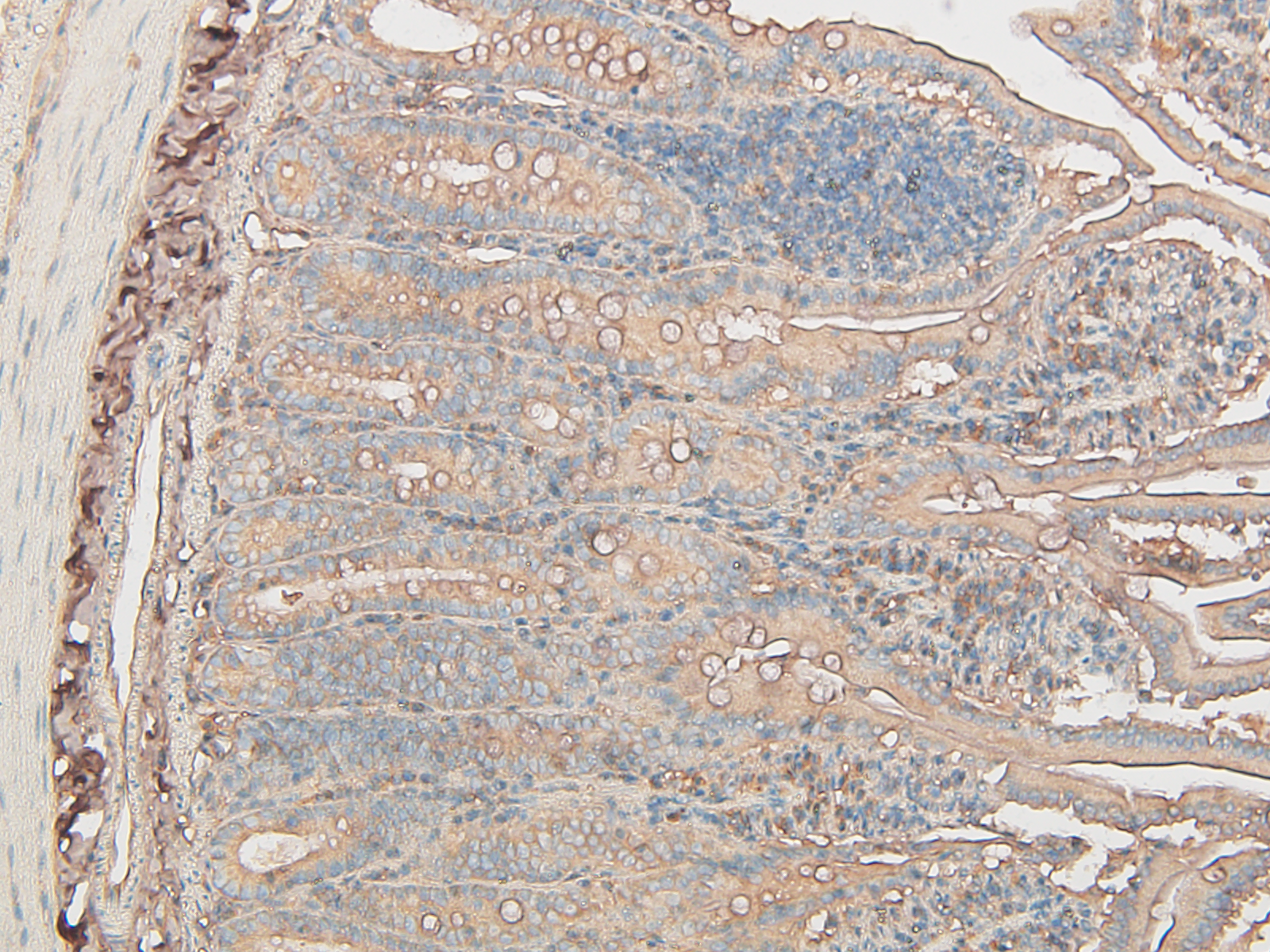

Supplement: Supplementary file 7 — Source data Fig. 5 [file 44318_2024_300_MOESM7_ESM.zip › Figure 5/fig5b/KO-MC4R-Nesfatin-1.tif]

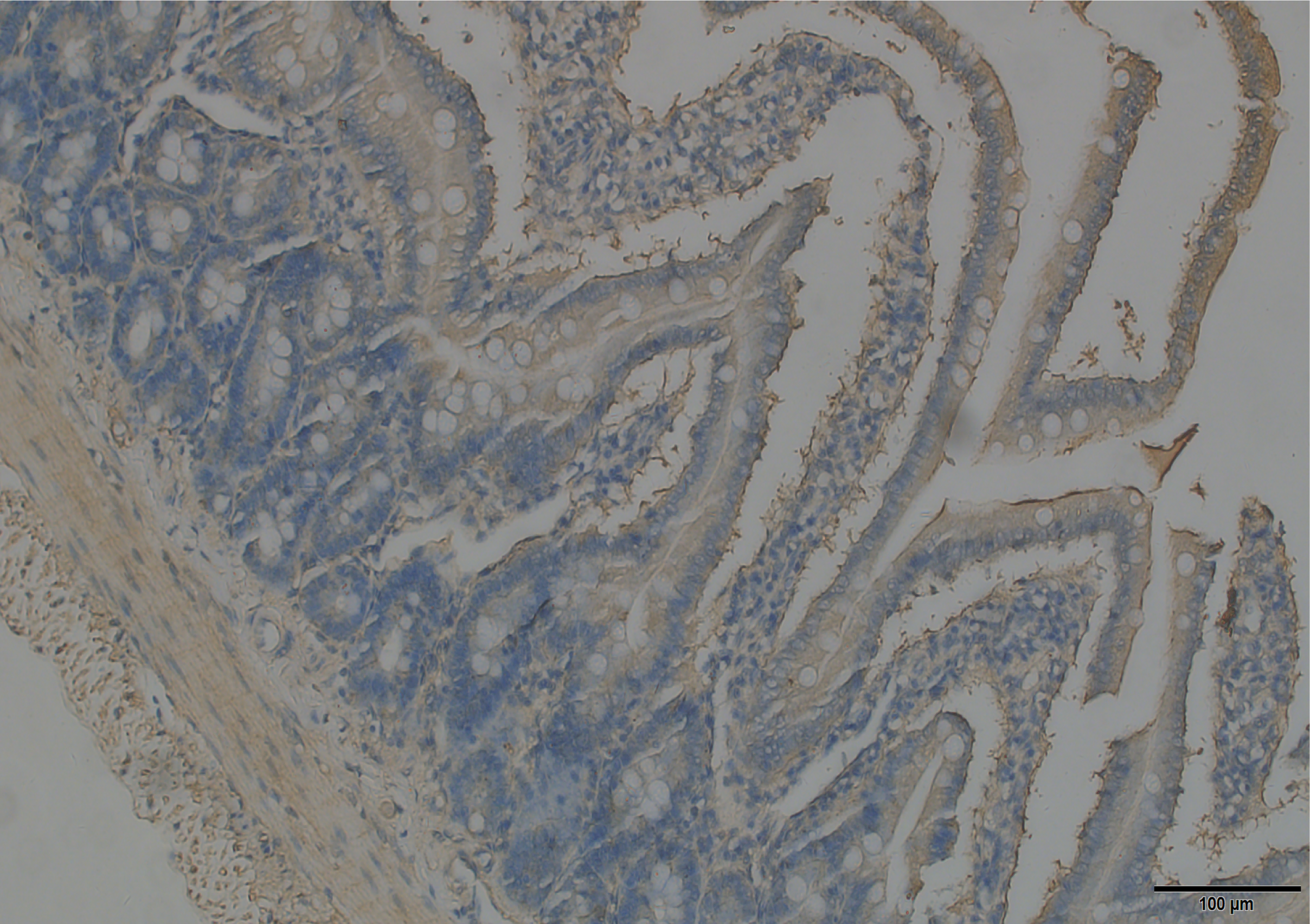

Supplement: Supplementary file 7 — Source data Fig. 5 [file 44318_2024_300_MOESM7_ESM.zip › Figure 5/fig5b/KO-MC4R-Saline.tif]

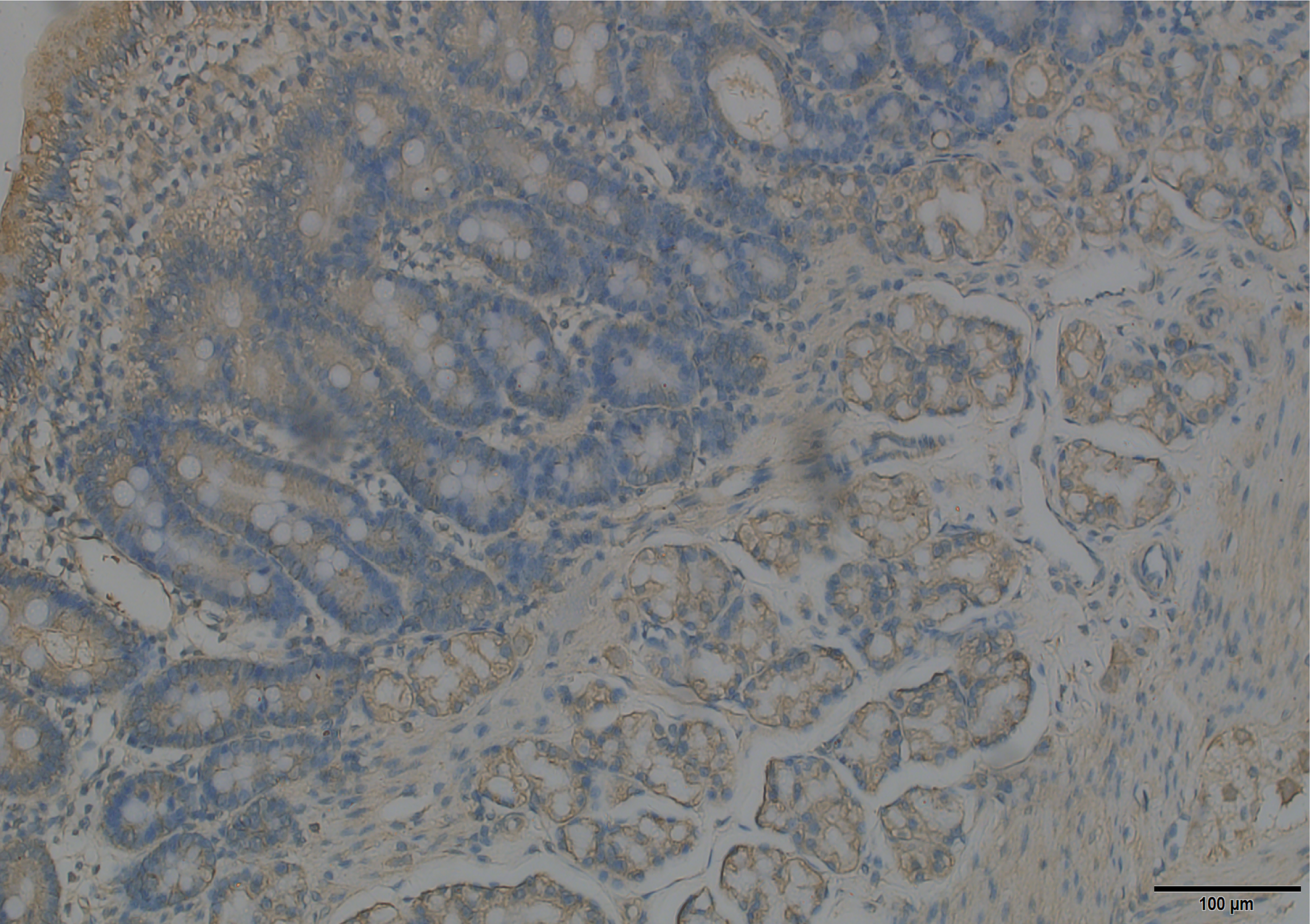

Supplement: Supplementary file 7 — Source data Fig. 5 [file 44318_2024_300_MOESM7_ESM.zip › Figure 5/fig5b/KO-NUCB2-Nesfatin-1.tif]

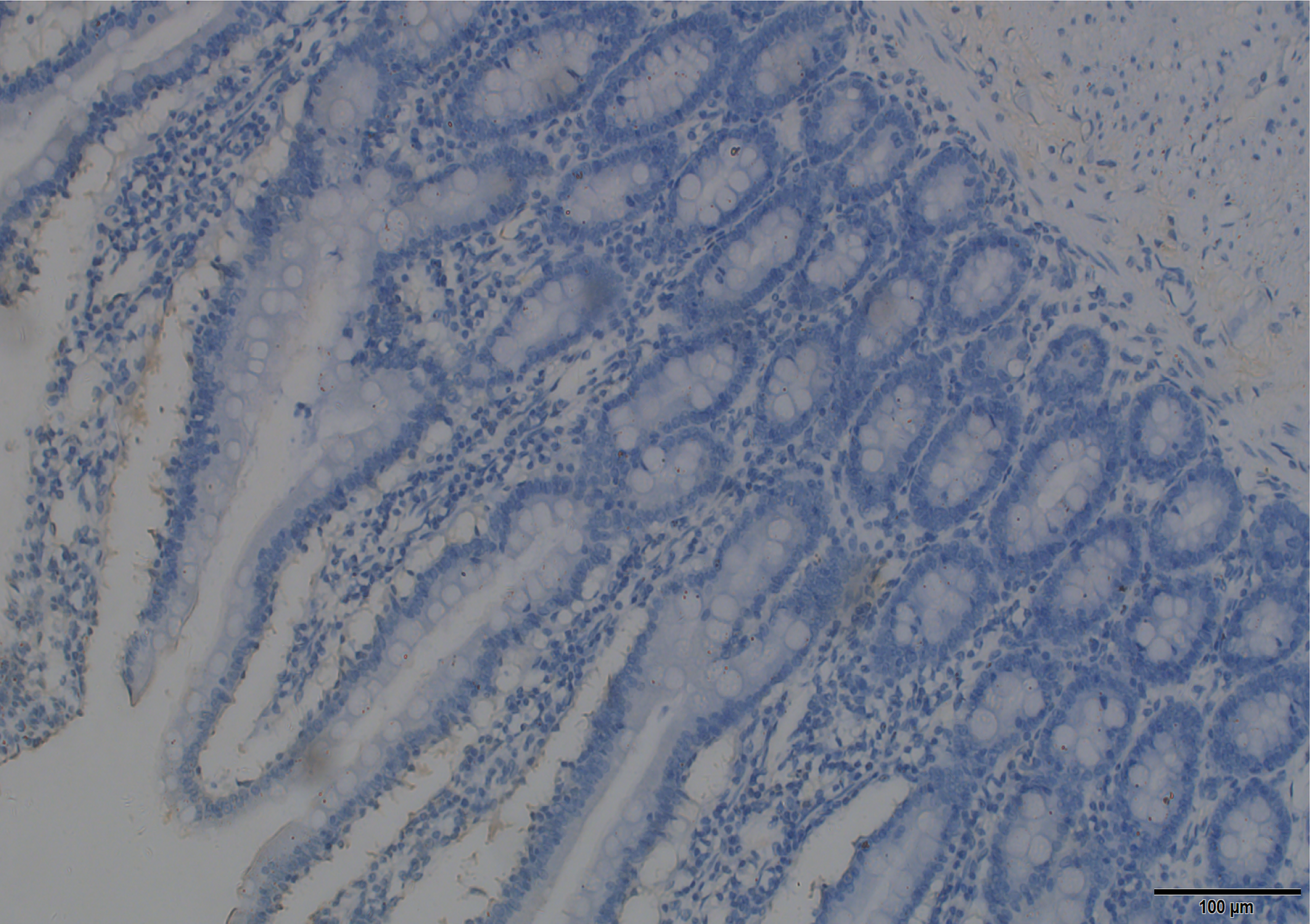

Supplement: Supplementary file 7 — Source data Fig. 5 [file 44318_2024_300_MOESM7_ESM.zip › Figure 5/fig5b/KO-NUCB2-Saline.tif]

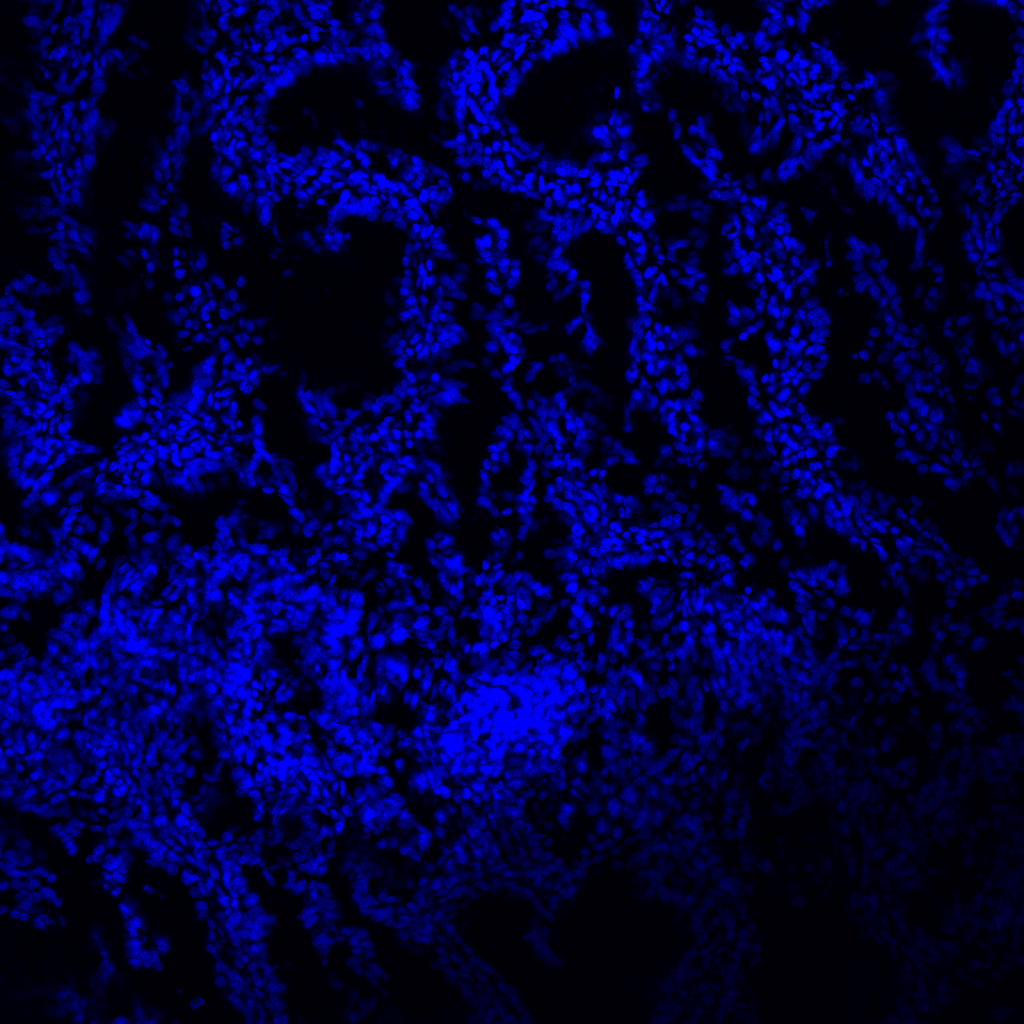

Supplement: Supplementary file 7 — Source data Fig. 5 [file 44318_2024_300_MOESM7_ESM.zip › Figure 5/fig5e/Nesfatin-1+SHU_DAPI.tif]

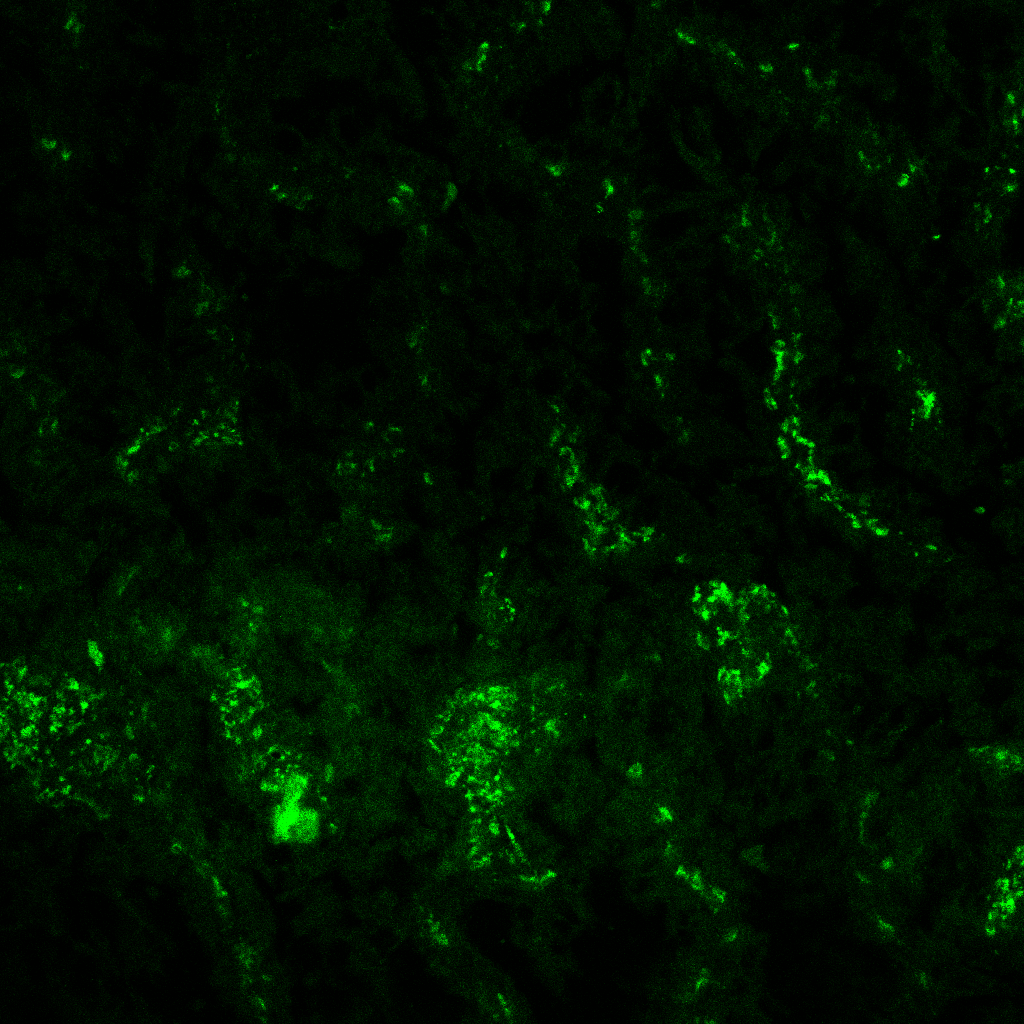

Supplement: Supplementary file 7 — Source data Fig. 5 [file 44318_2024_300_MOESM7_ESM.zip › Figure 5/fig5e/Nesfatin-1+SHU_GLP-1.tif]

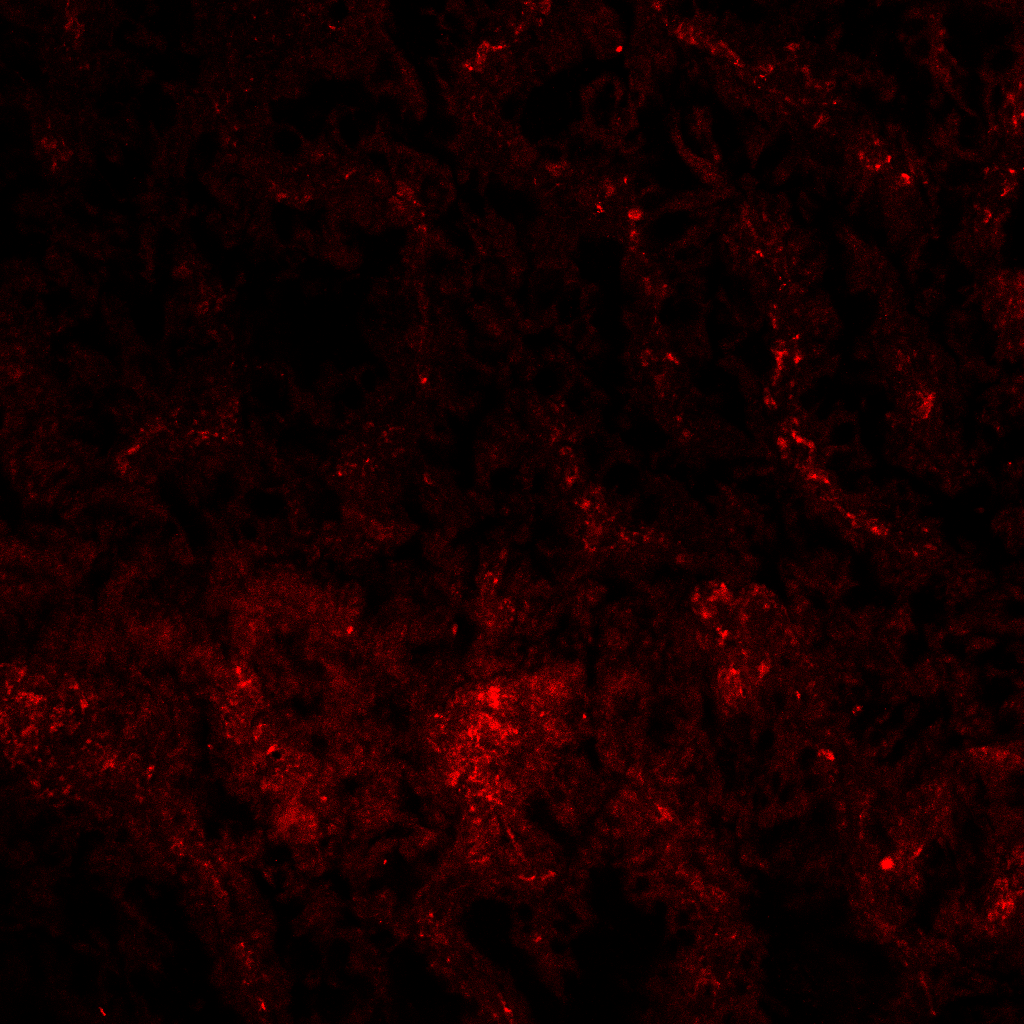

Supplement: Supplementary file 7 — Source data Fig. 5 [file 44318_2024_300_MOESM7_ESM.zip › Figure 5/fig5e/Nesfatin-1+SHU_MC4R.tif]

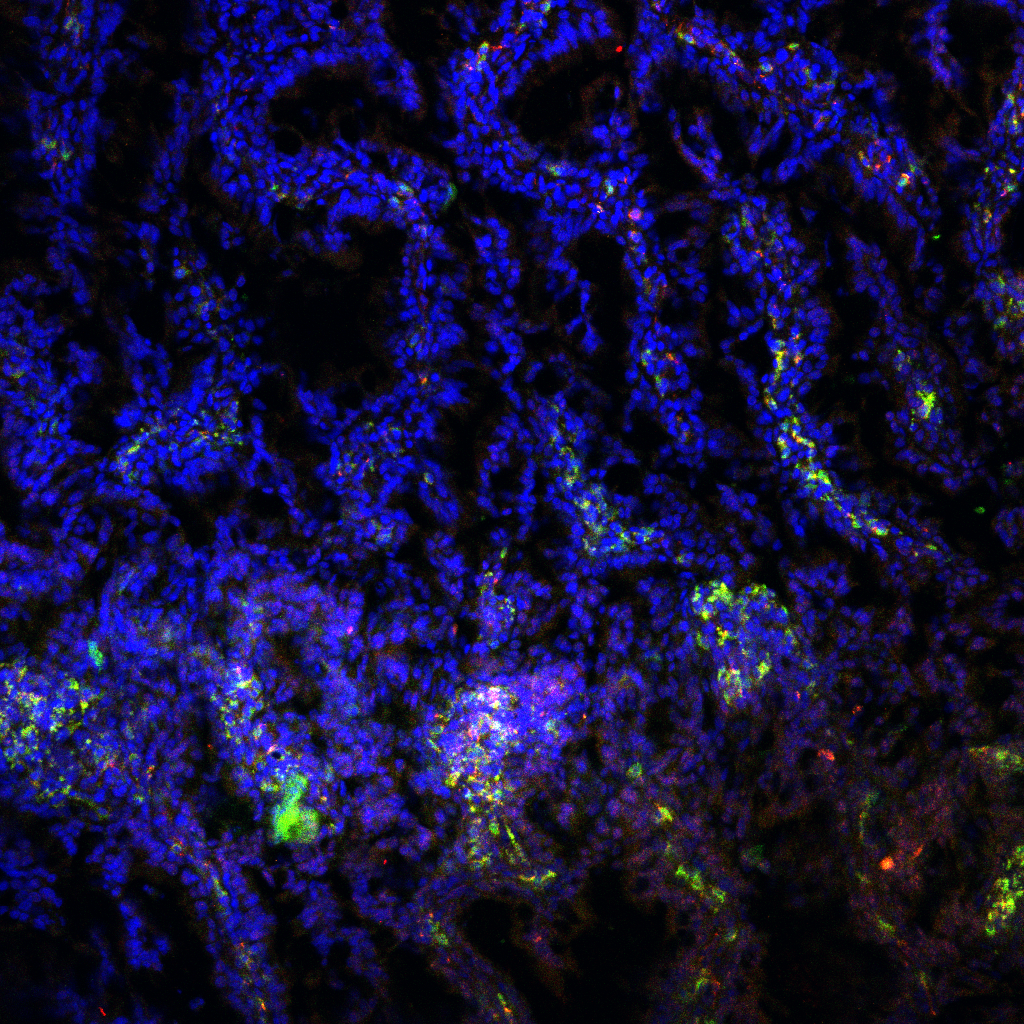

Supplement: Supplementary file 7 — Source data Fig. 5 [file 44318_2024_300_MOESM7_ESM.zip › Figure 5/fig5e/Nesfatin-1+SHU_Merged.tif]

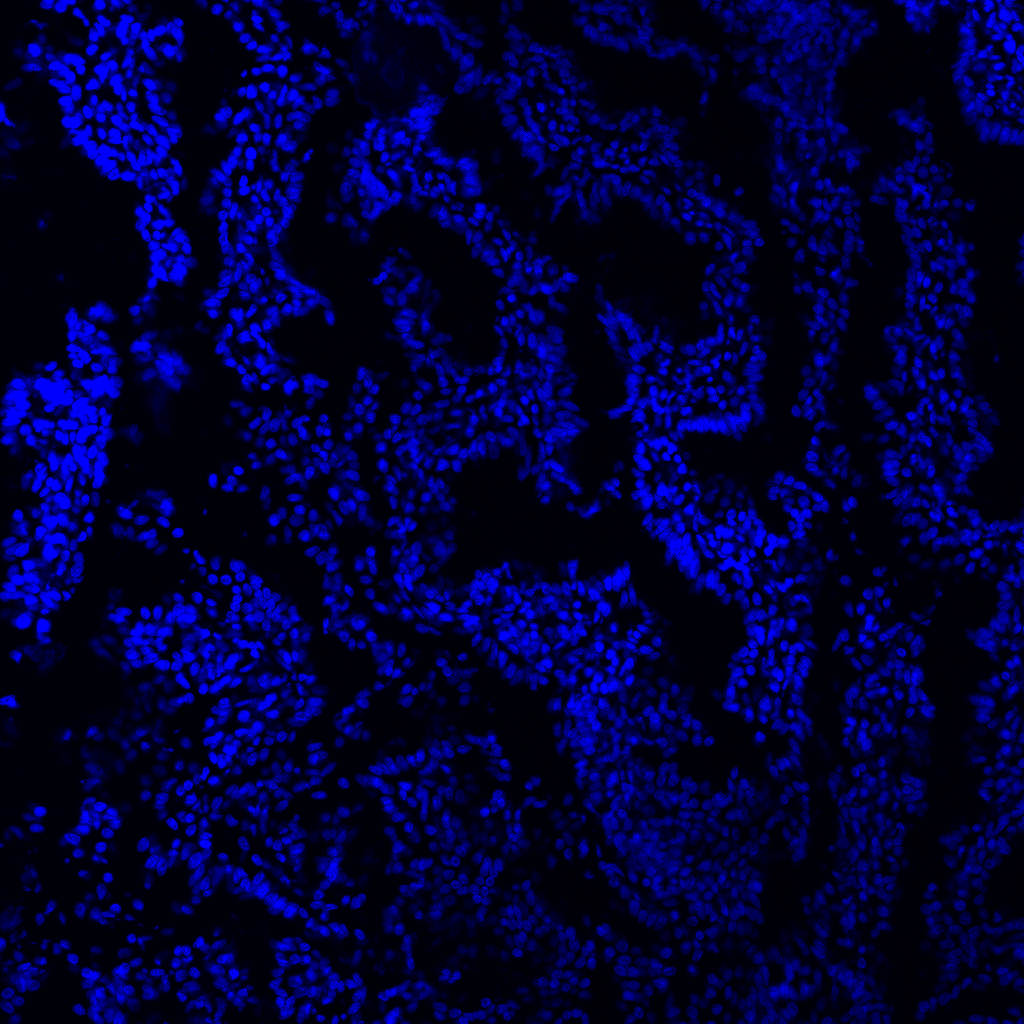

Supplement: Supplementary file 7 — Source data Fig. 5 [file 44318_2024_300_MOESM7_ESM.zip › Figure 5/fig5e/Nesfatin-1_DAPI.tif]

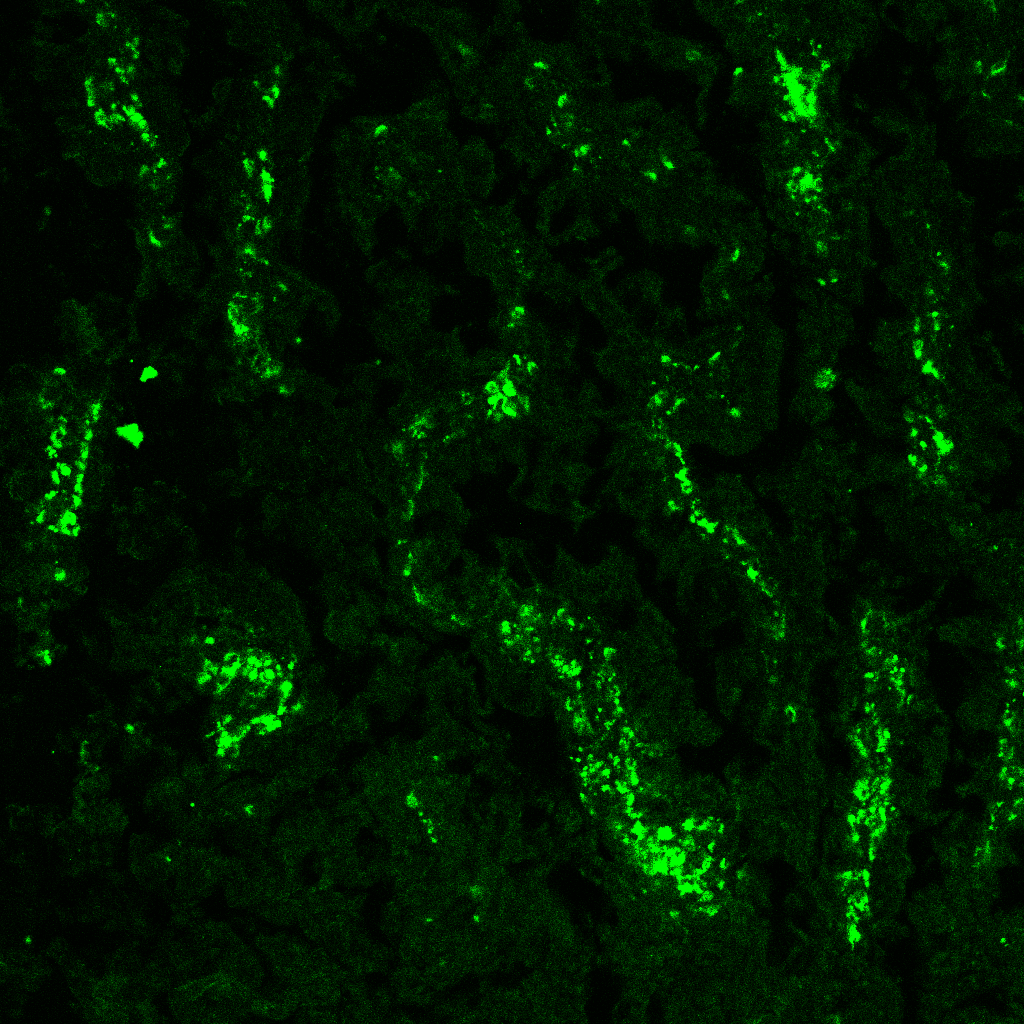

Supplement: Supplementary file 7 — Source data Fig. 5 [file 44318_2024_300_MOESM7_ESM.zip › Figure 5/fig5e/Nesfatin-1_GLP-1.tif]

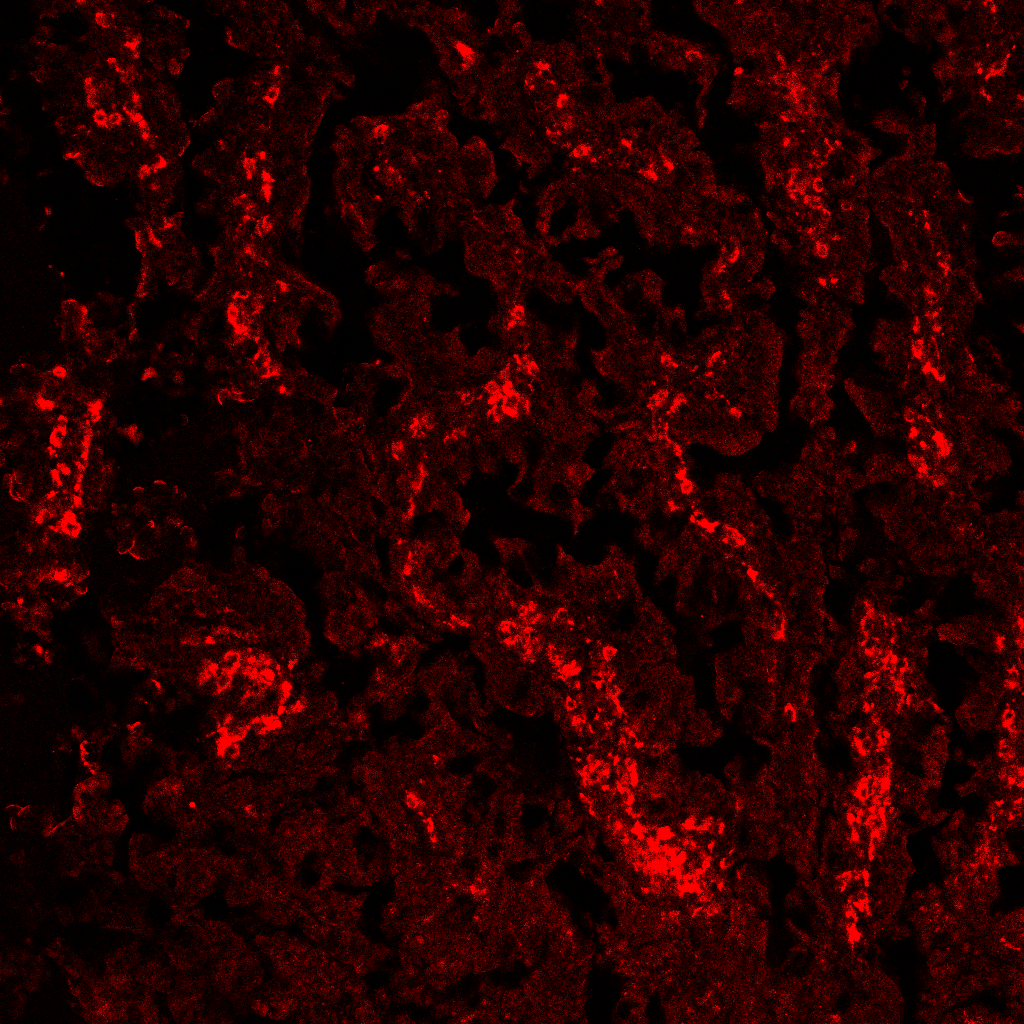

Supplement: Supplementary file 7 — Source data Fig. 5 [file 44318_2024_300_MOESM7_ESM.zip › Figure 5/fig5e/Nesfatin-1_MC4R.tif]

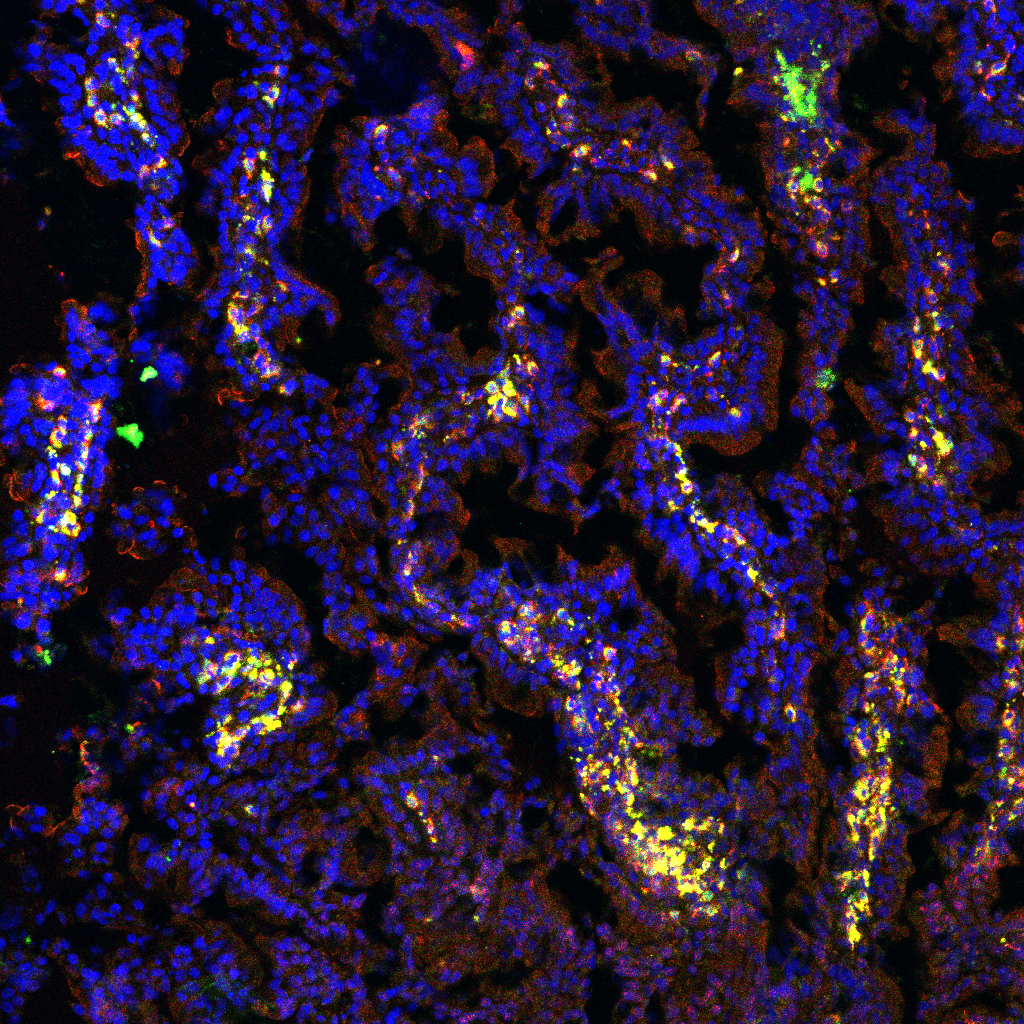

Supplement: Supplementary file 7 — Source data Fig. 5 [file 44318_2024_300_MOESM7_ESM.zip › Figure 5/fig5e/Nesfatin-1_Merged.tif]

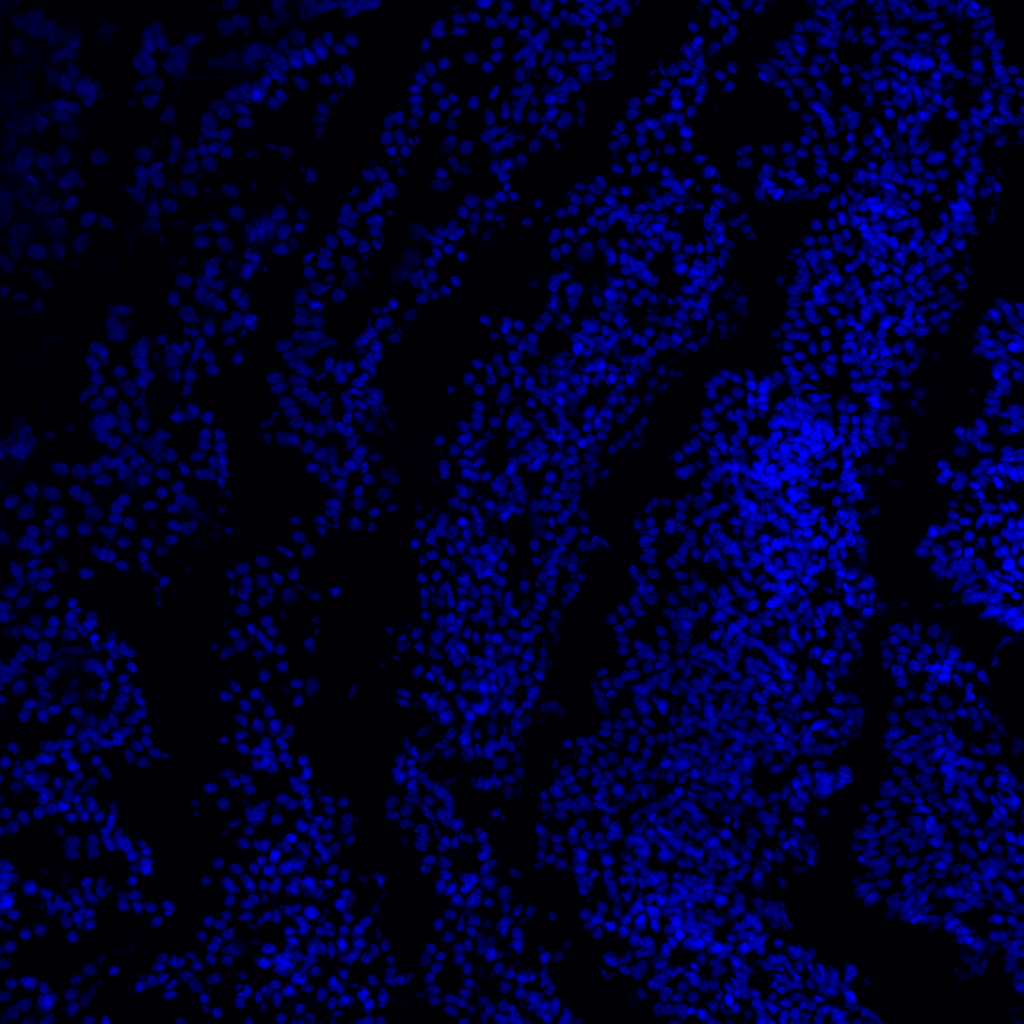

Supplement: Supplementary file 7 — Source data Fig. 5 [file 44318_2024_300_MOESM7_ESM.zip › Figure 5/fig5e/Saline_DAPI.tif]

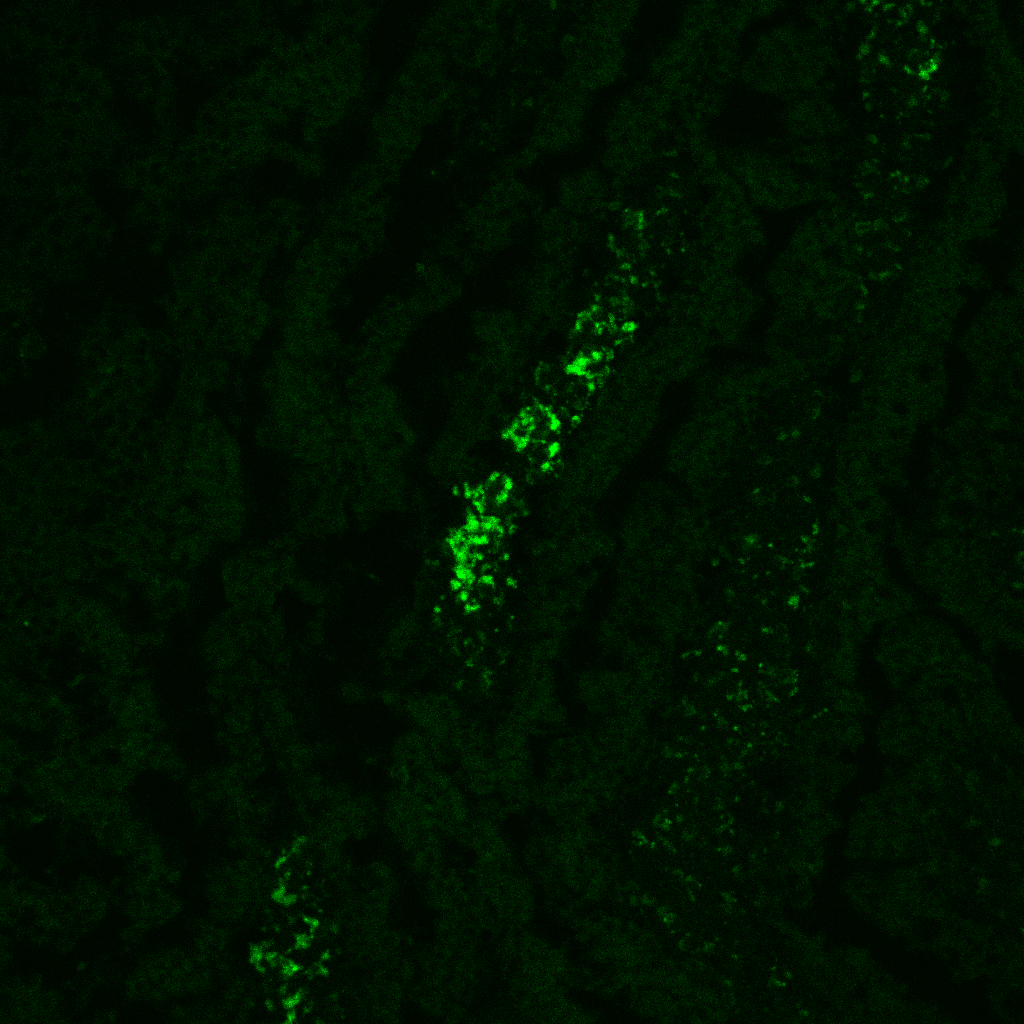

Supplement: Supplementary file 7 — Source data Fig. 5 [file 44318_2024_300_MOESM7_ESM.zip › Figure 5/fig5e/Saline_GLP-1.tif]

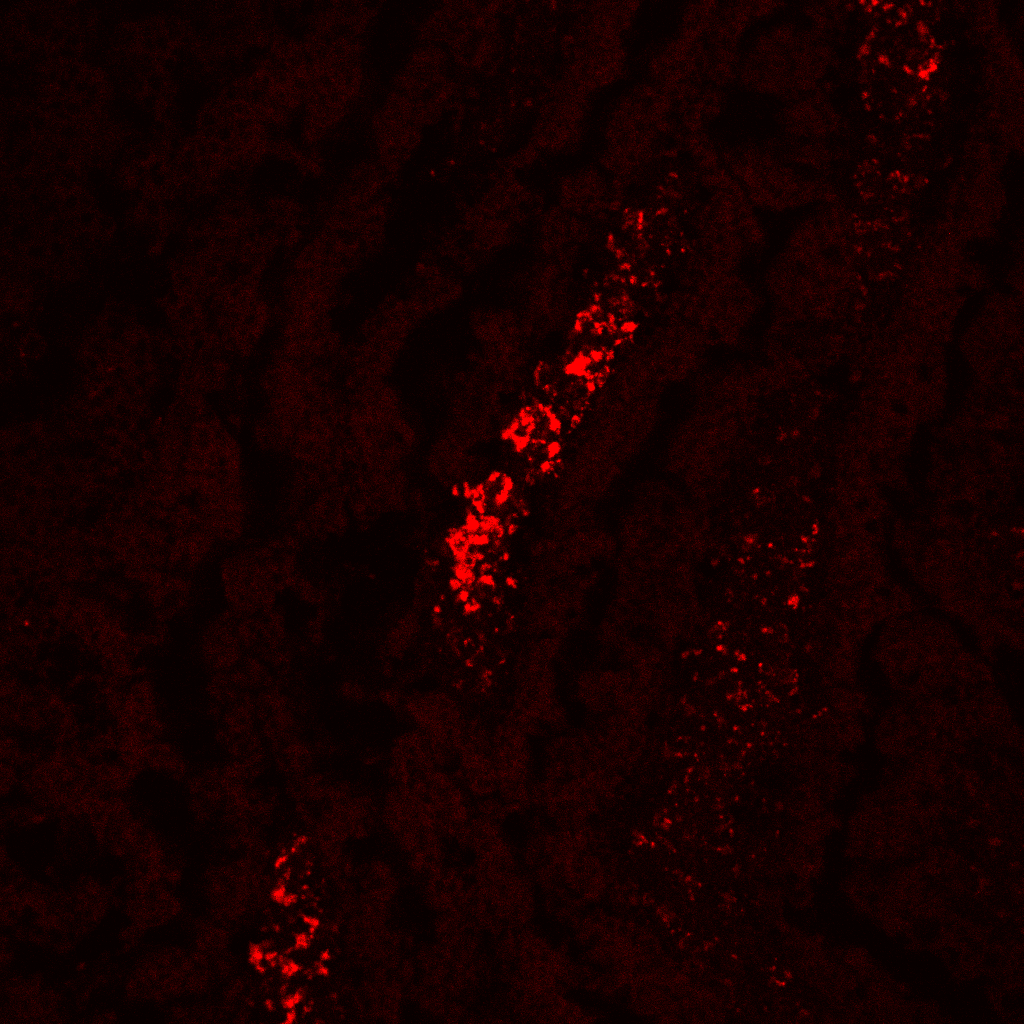

Supplement: Supplementary file 7 — Source data Fig. 5 [file 44318_2024_300_MOESM7_ESM.zip › Figure 5/fig5e/Saline_MC4R.tif]

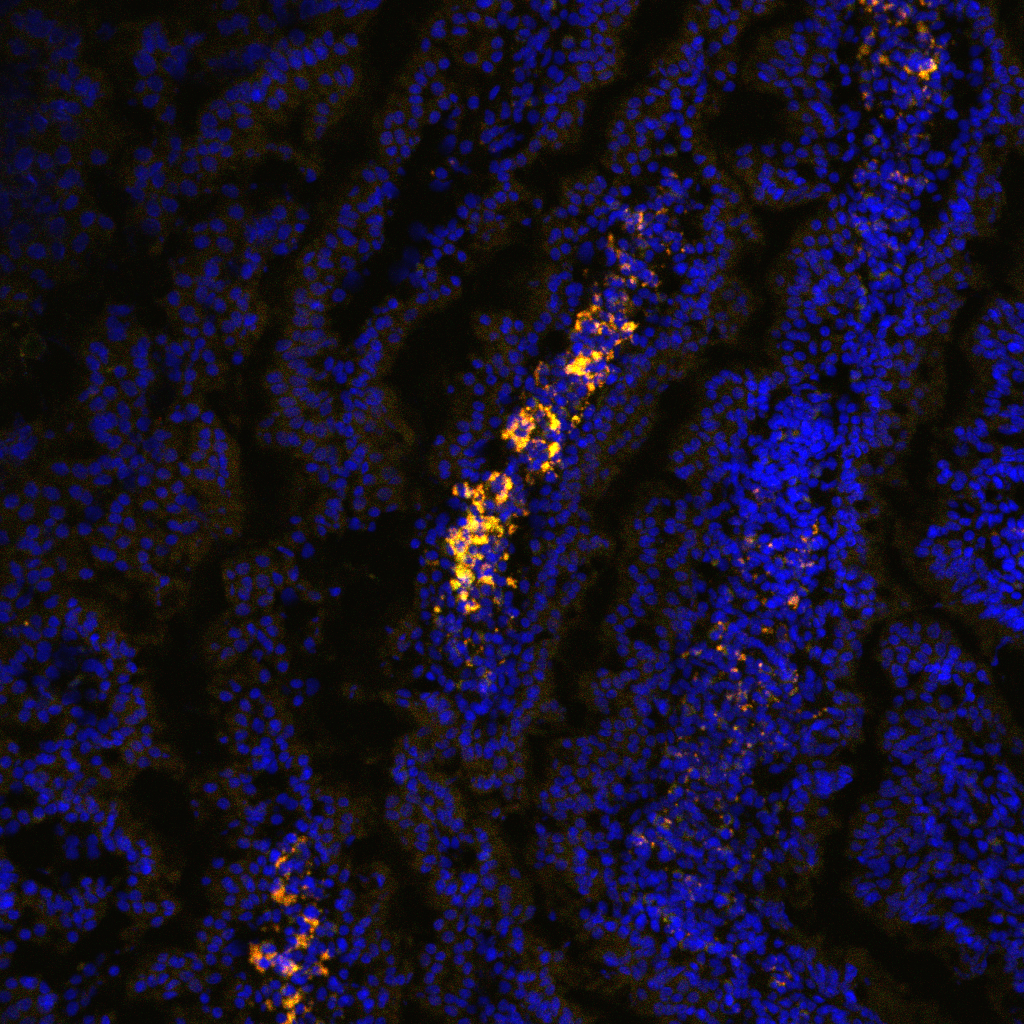

Supplement: Supplementary file 7 — Source data Fig. 5 [file 44318_2024_300_MOESM7_ESM.zip › Figure 5/fig5e/Saline_Merged.tif]

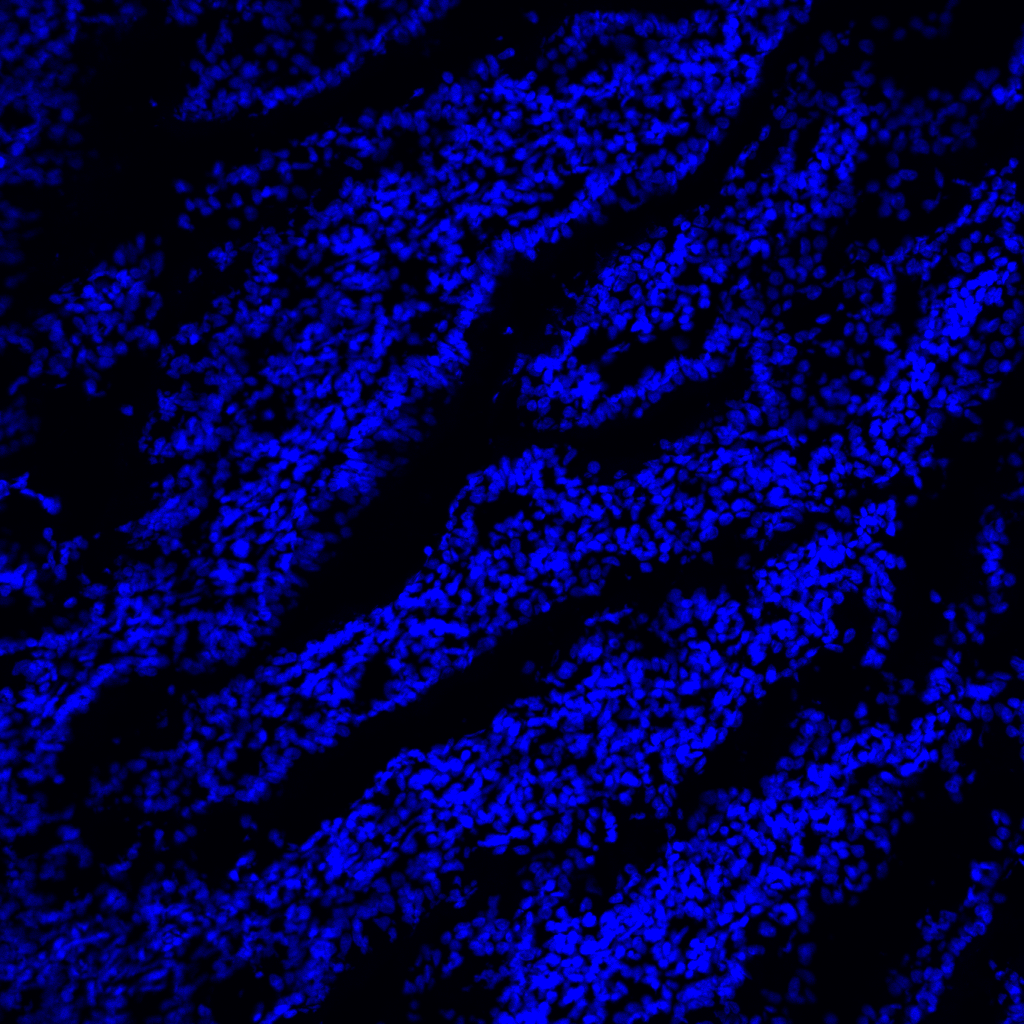

Supplement: Supplementary file 7 — Source data Fig. 5 [file 44318_2024_300_MOESM7_ESM.zip › Figure 5/fig5e/SHU_DAPI.tif]

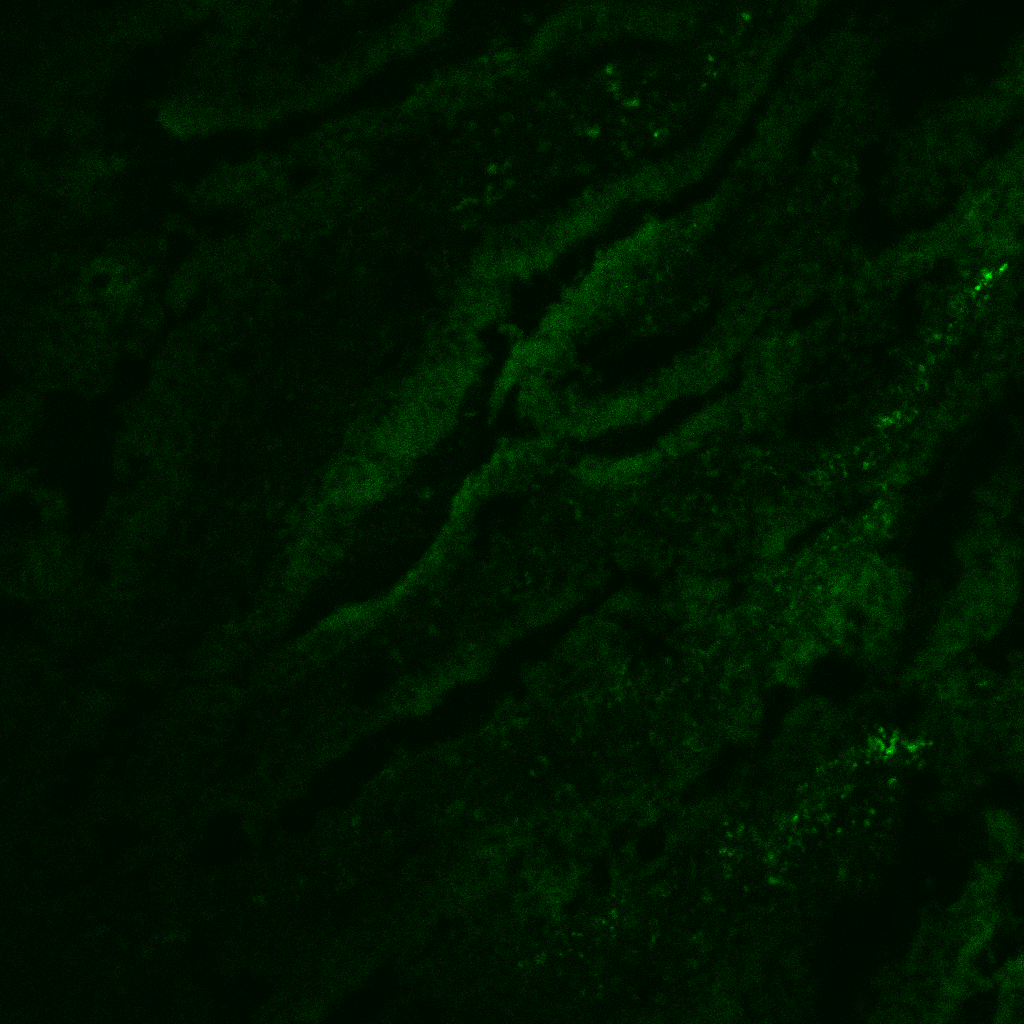

Supplement: Supplementary file 7 — Source data Fig. 5 [file 44318_2024_300_MOESM7_ESM.zip › Figure 5/fig5e/SHU_GLP-1.tif]

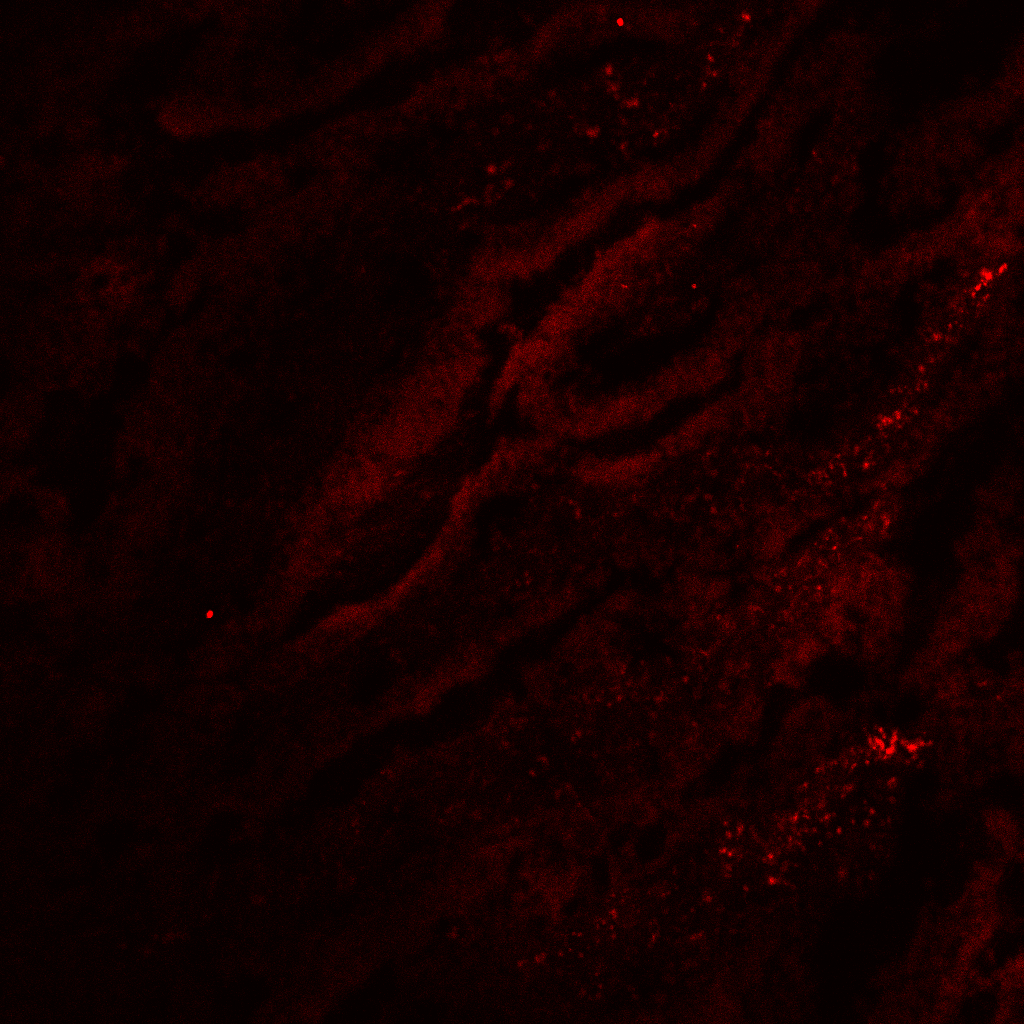

Supplement: Supplementary file 7 — Source data Fig. 5 [file 44318_2024_300_MOESM7_ESM.zip › Figure 5/fig5e/SHU_MC4R.tif]

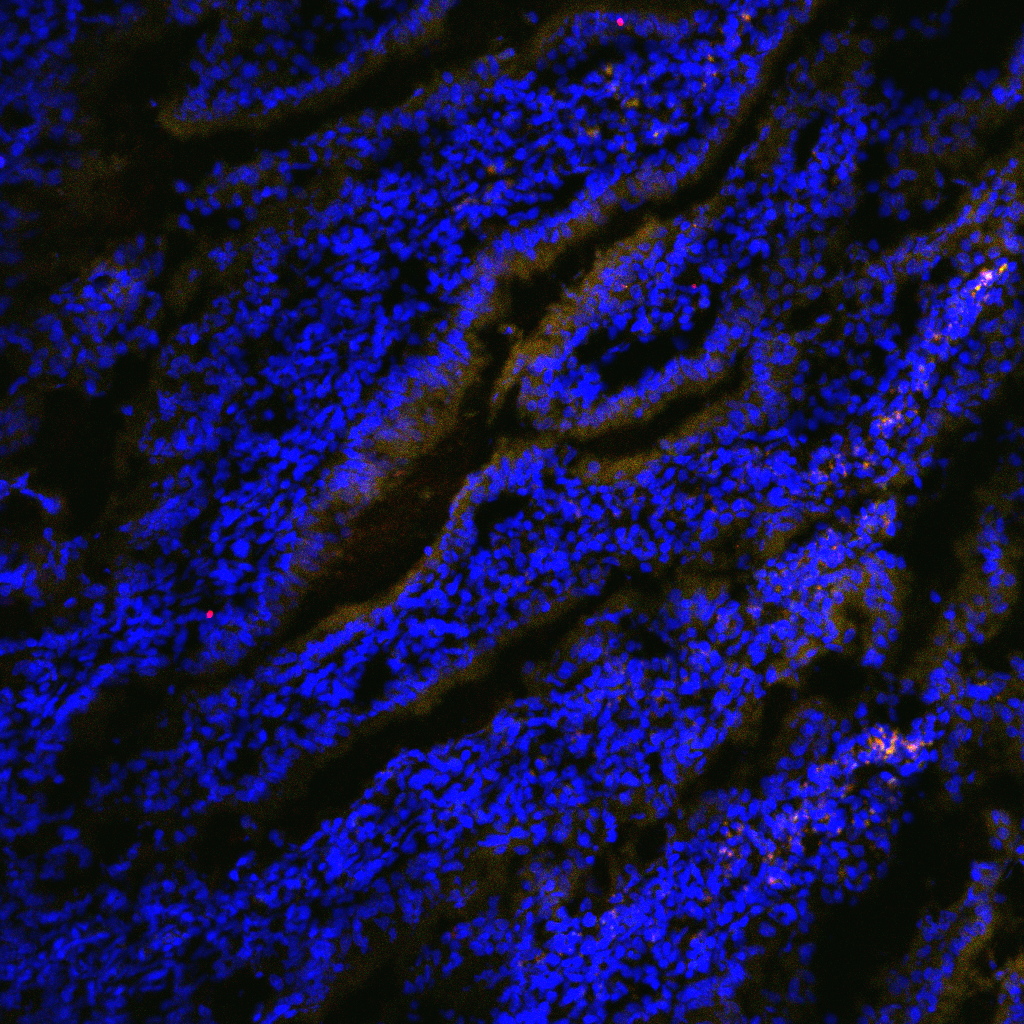

Supplement: Supplementary file 7 — Source data Fig. 5 [file 44318_2024_300_MOESM7_ESM.zip › Figure 5/fig5e/SHU_Merged.tif]

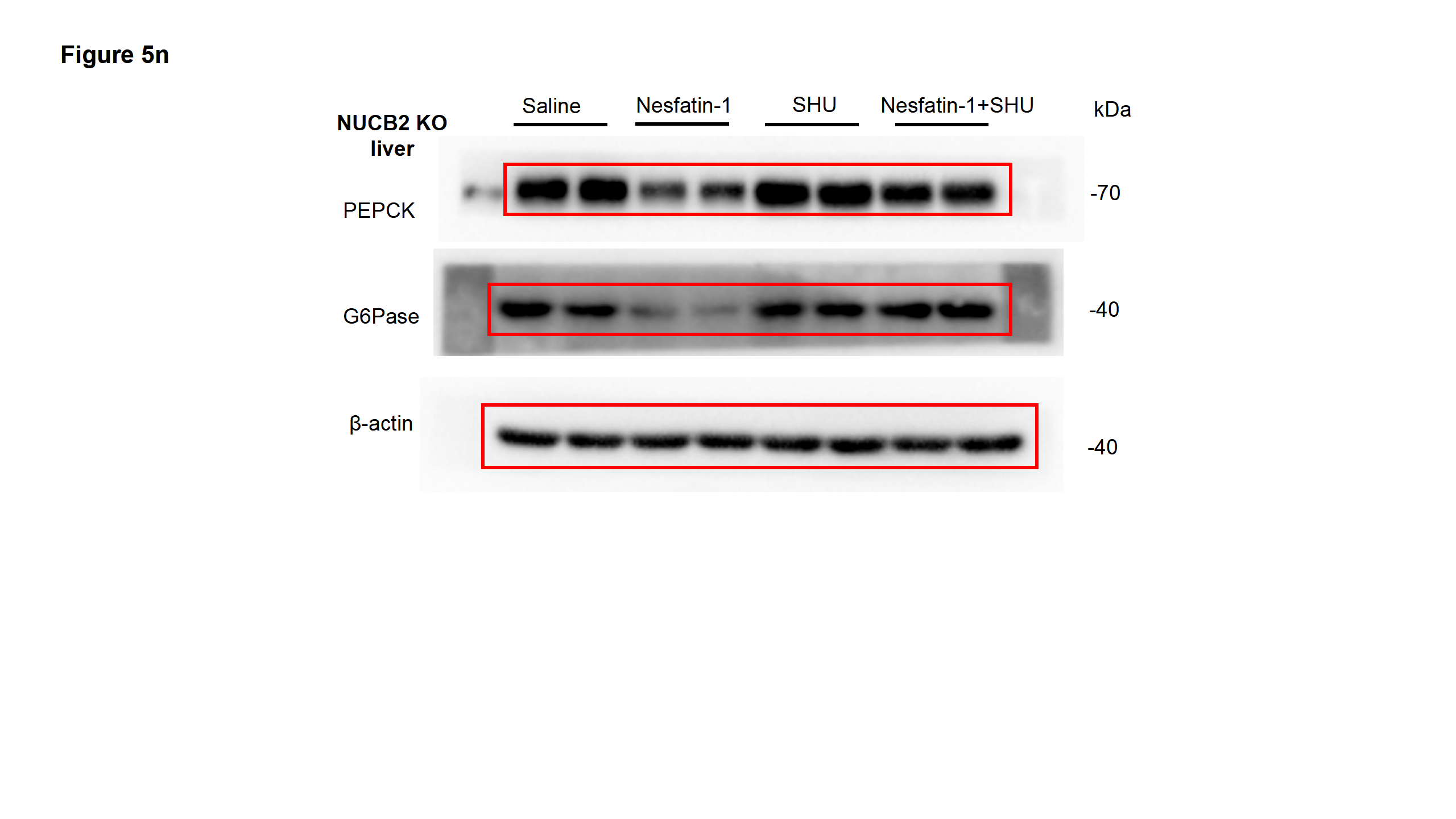

Supplement: Supplementary file 7 — Source data Fig. 5 [file 44318_2024_300_MOESM7_ESM.zip › Figure 5/fig5n/fig5n.tif]

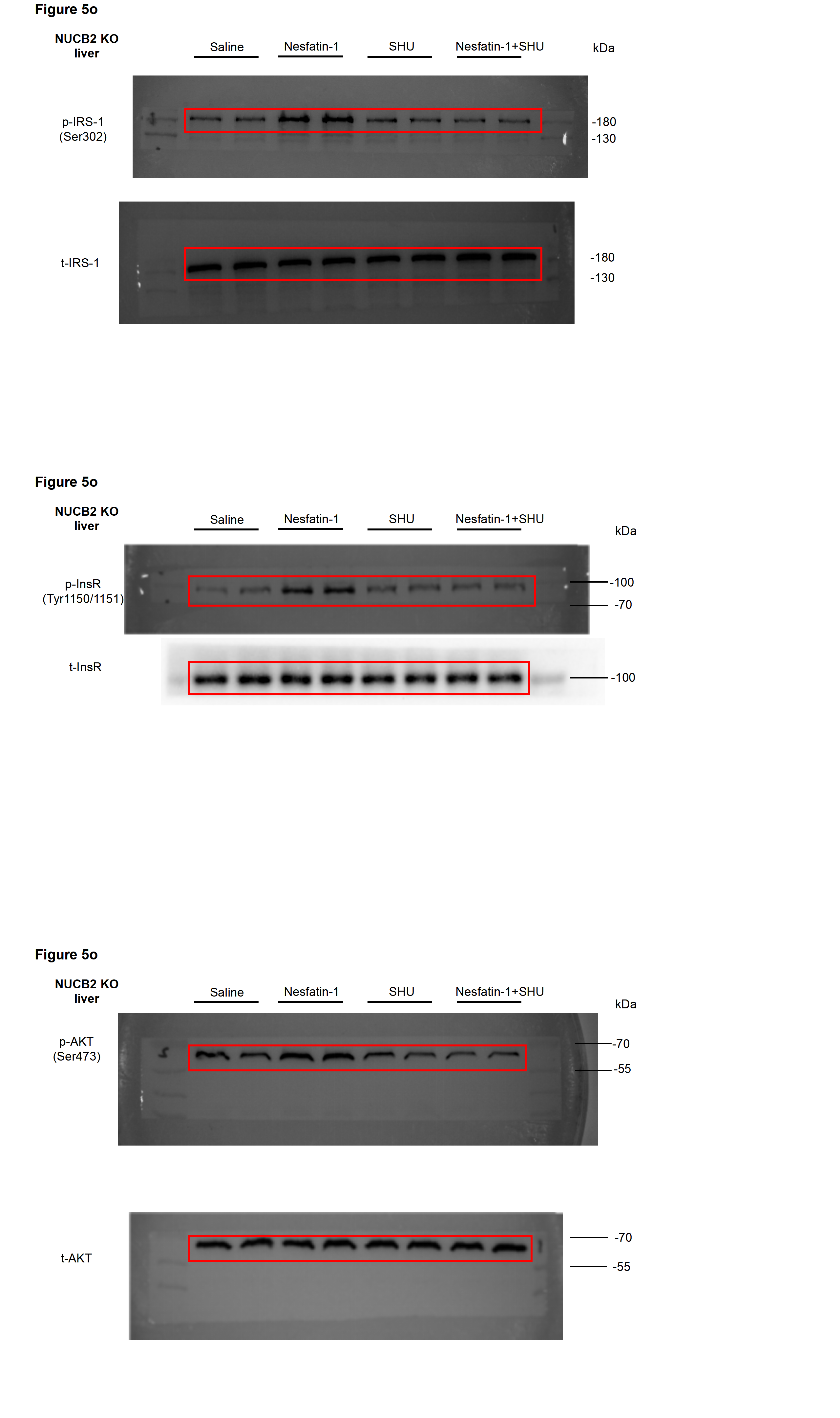

Supplement: Supplementary file 7 — Source data Fig. 5 [file 44318_2024_300_MOESM7_ESM.zip › Figure 5/fig5o/fig5o.tif]

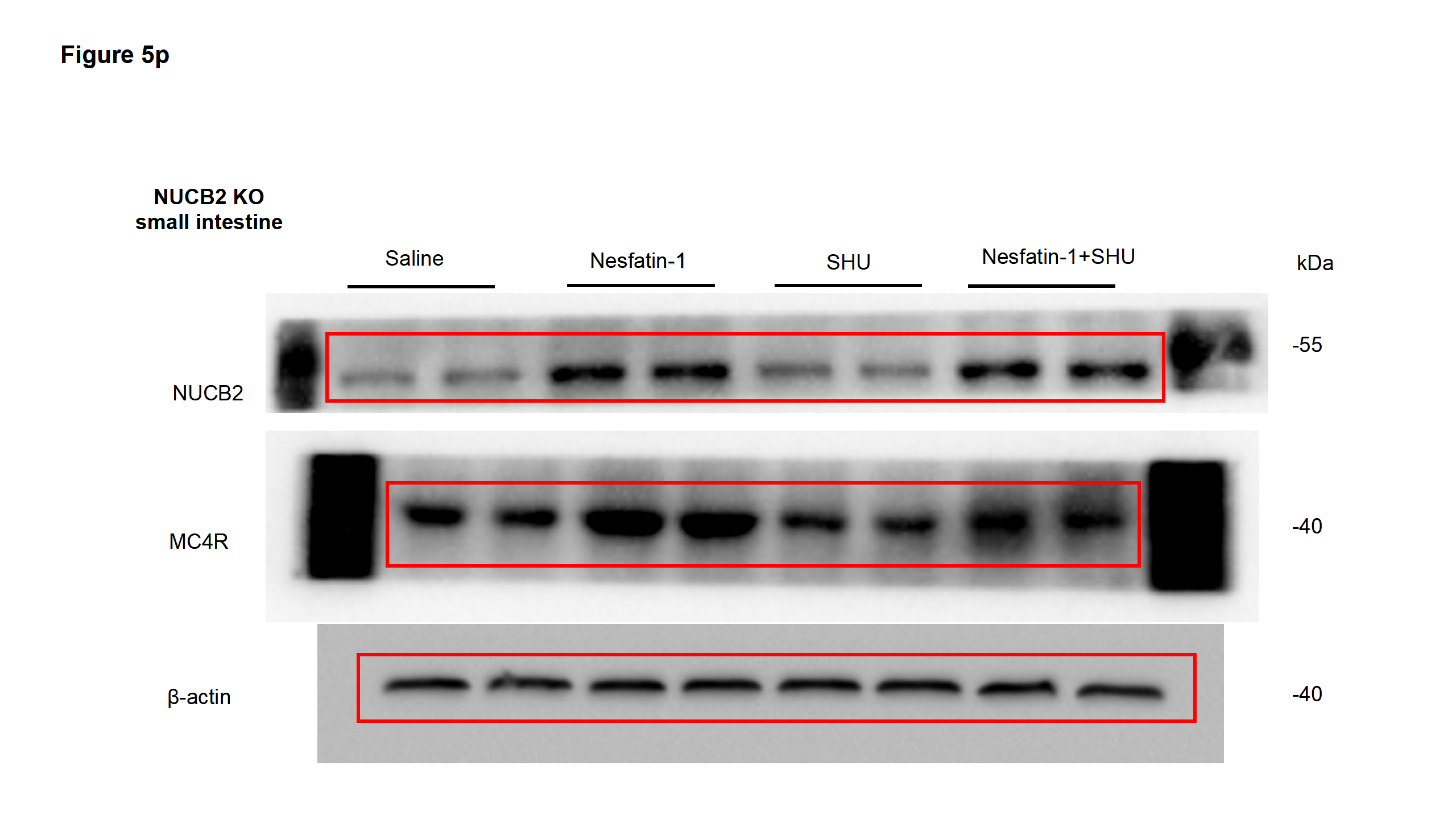

Supplement: Supplementary file 7 — Source data Fig. 5 [file 44318_2024_300_MOESM7_ESM.zip › Figure 5/fig5p/fig5p.tif]

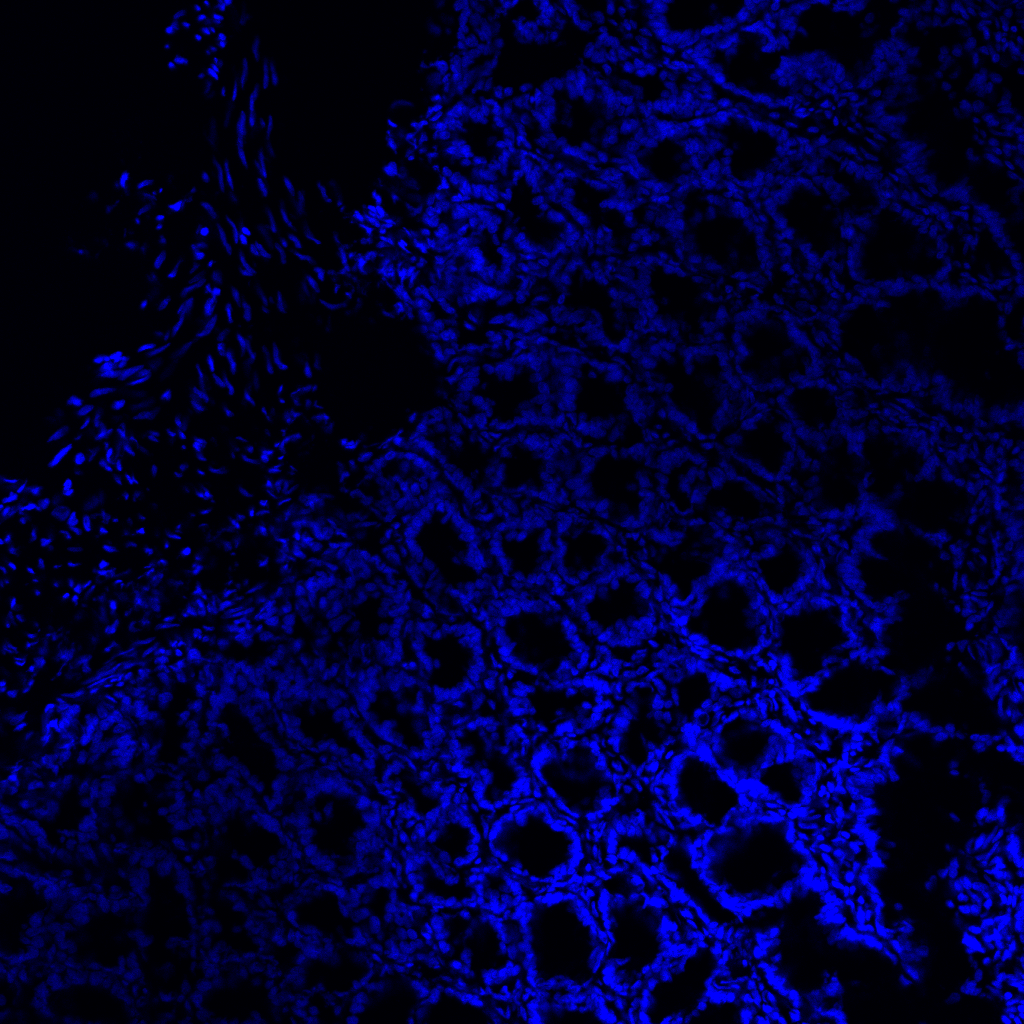

Supplement: Supplementary file 8 — Source data Fig. 6 [file 44318_2024_300_MOESM8_ESM.zip › Figure 6/fig6c/100μm_DAPI.tif]

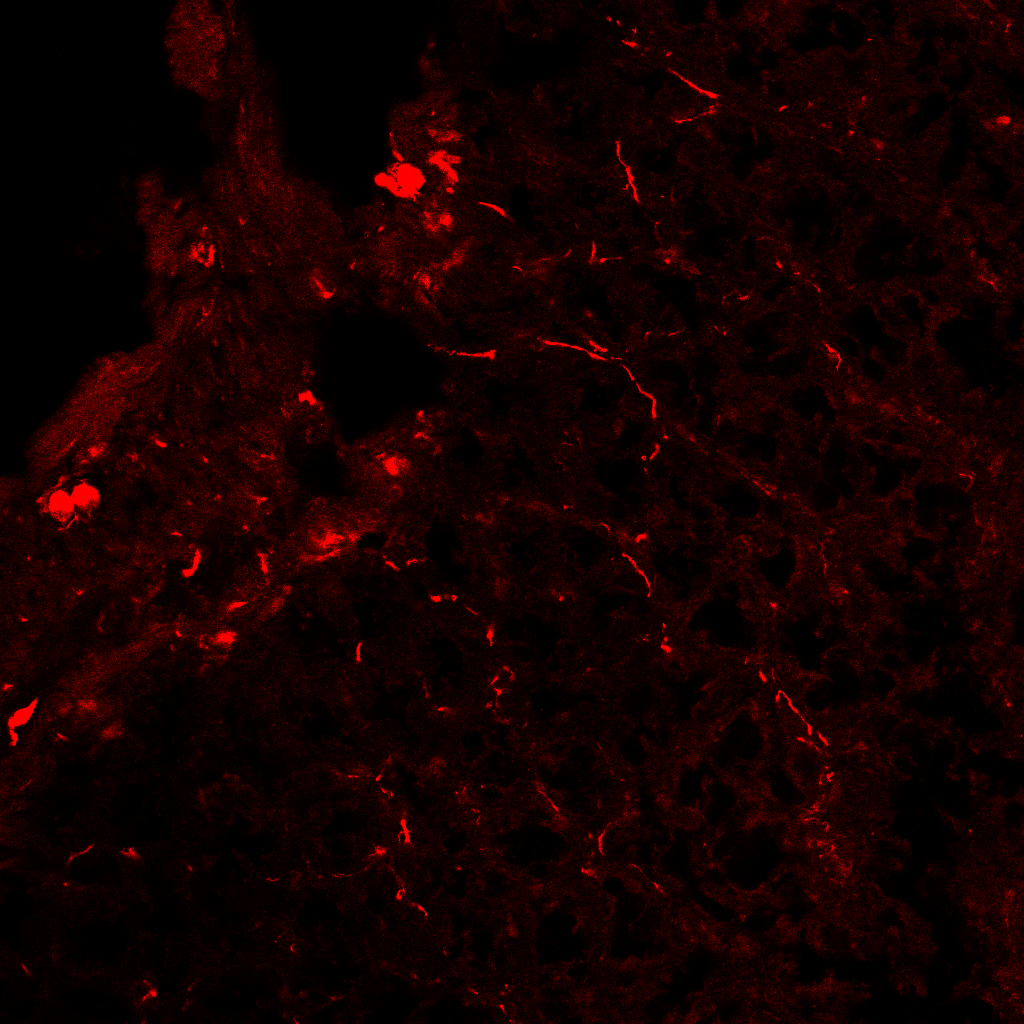

Supplement: Supplementary file 8 — Source data Fig. 6 [file 44318_2024_300_MOESM8_ESM.zip › Figure 6/fig6c/100μm_MC4R.tif]

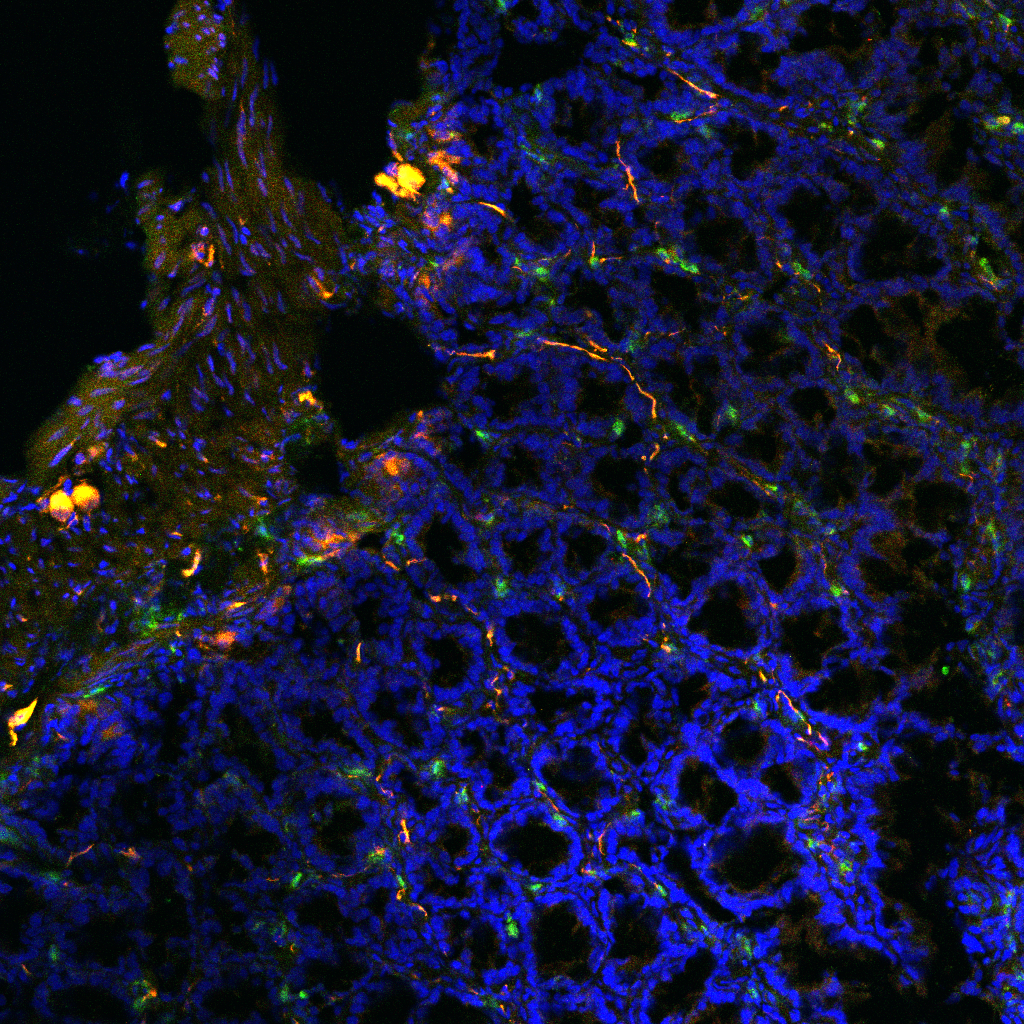

Supplement: Supplementary file 8 — Source data Fig. 6 [file 44318_2024_300_MOESM8_ESM.zip › Figure 6/fig6c/100μm_merged.tif]

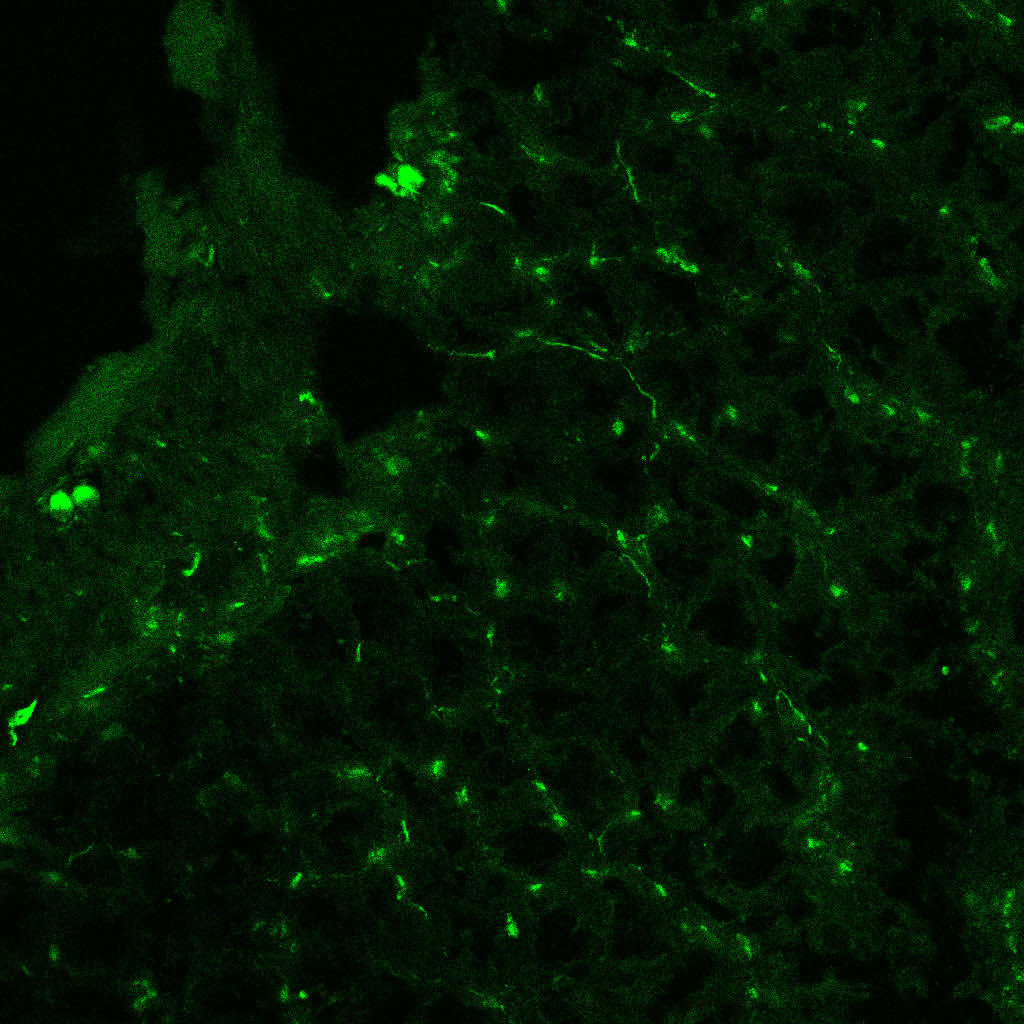

Supplement: Supplementary file 8 — Source data Fig. 6 [file 44318_2024_300_MOESM8_ESM.zip › Figure 6/fig6c/100μm_NUCB2.tif]

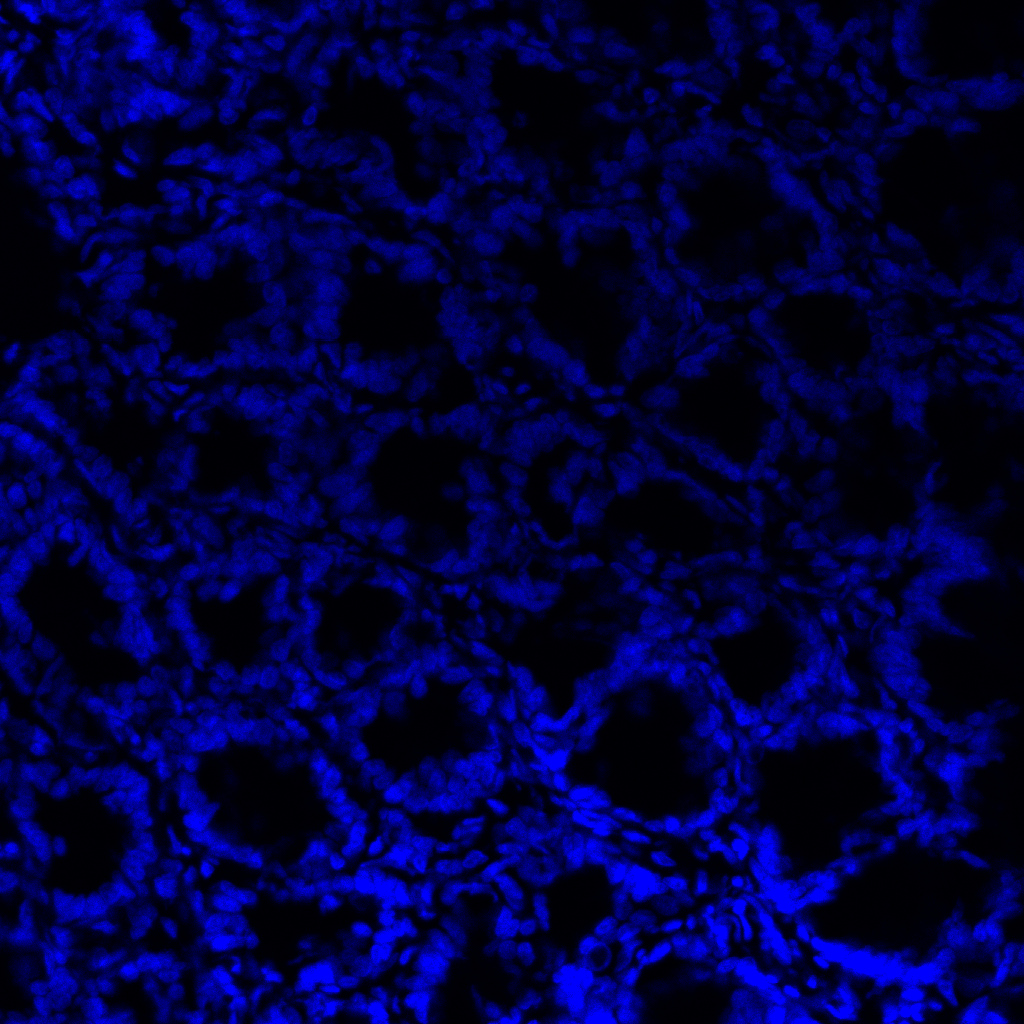

Supplement: Supplementary file 8 — Source data Fig. 6 [file 44318_2024_300_MOESM8_ESM.zip › Figure 6/fig6c/60μm_DAPI.tif]

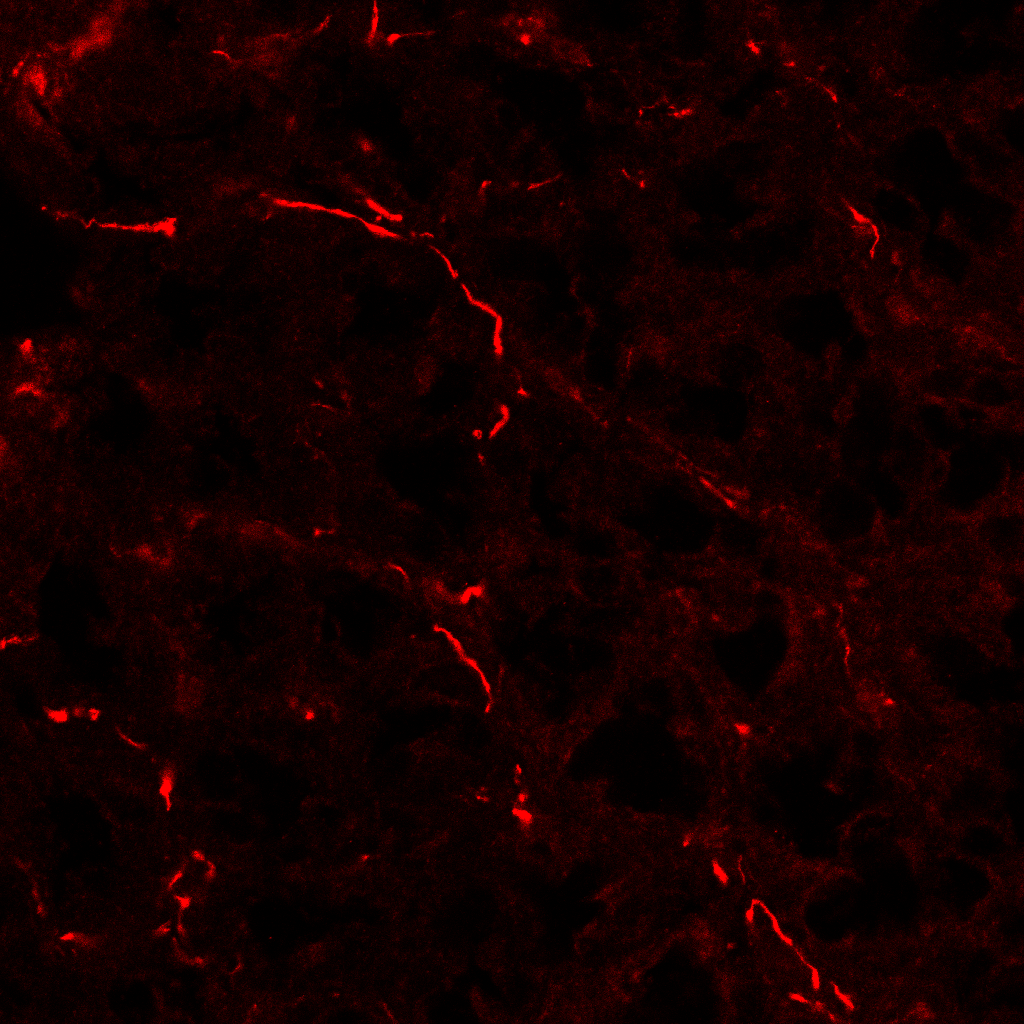

Supplement: Supplementary file 8 — Source data Fig. 6 [file 44318_2024_300_MOESM8_ESM.zip › Figure 6/fig6c/60μm_MC4R.tif]

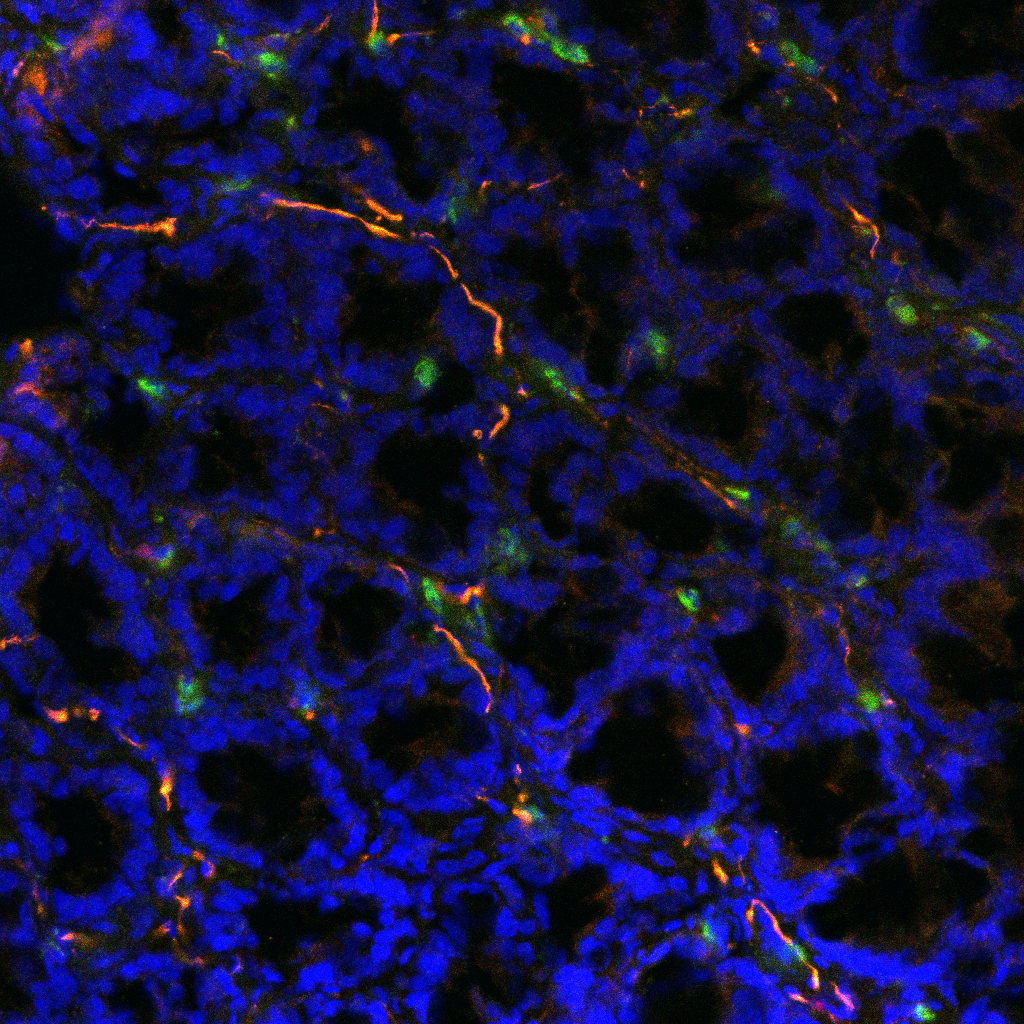

Supplement: Supplementary file 8 — Source data Fig. 6 [file 44318_2024_300_MOESM8_ESM.zip › Figure 6/fig6c/60μm_Merged.tif]

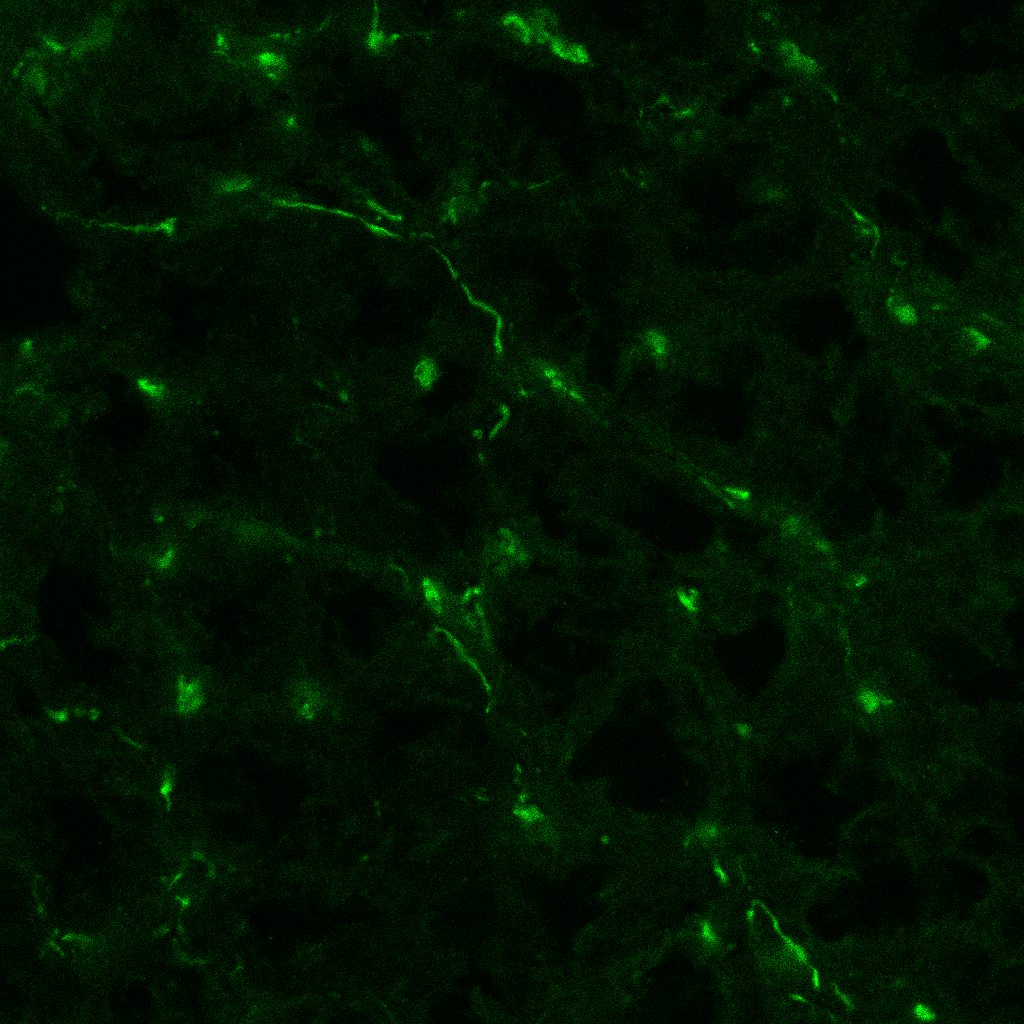

Supplement: Supplementary file 8 — Source data Fig. 6 [file 44318_2024_300_MOESM8_ESM.zip › Figure 6/fig6c/60μm_NUCB2.tif]

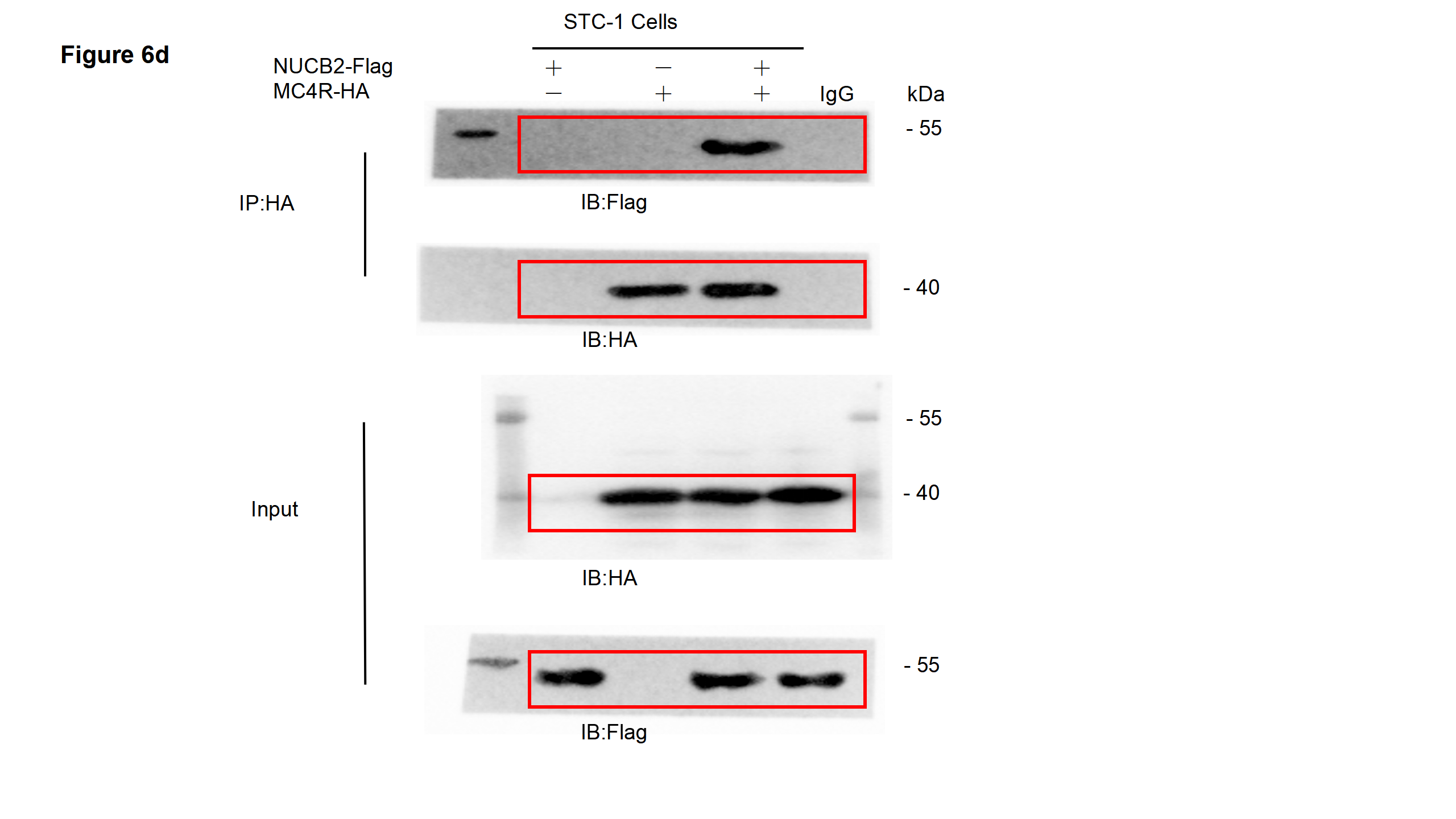

Supplement: Supplementary file 8 — Source data Fig. 6 [file 44318_2024_300_MOESM8_ESM.zip › Figure 6/fig6d/fig6d.tif]

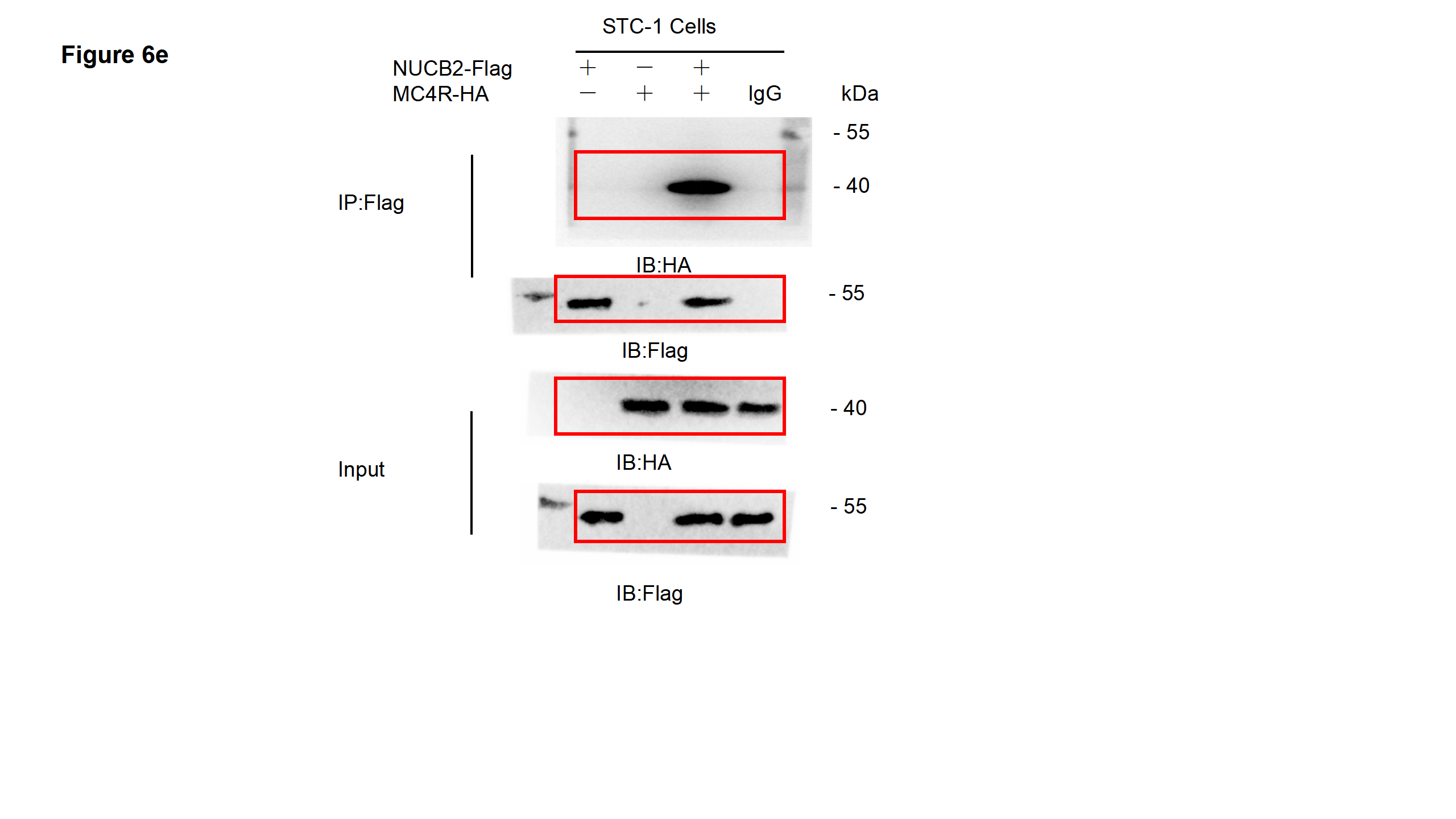

Supplement: Supplementary file 8 — Source data Fig. 6 [file 44318_2024_300_MOESM8_ESM.zip › Figure 6/fig6e/fig6e.tif]

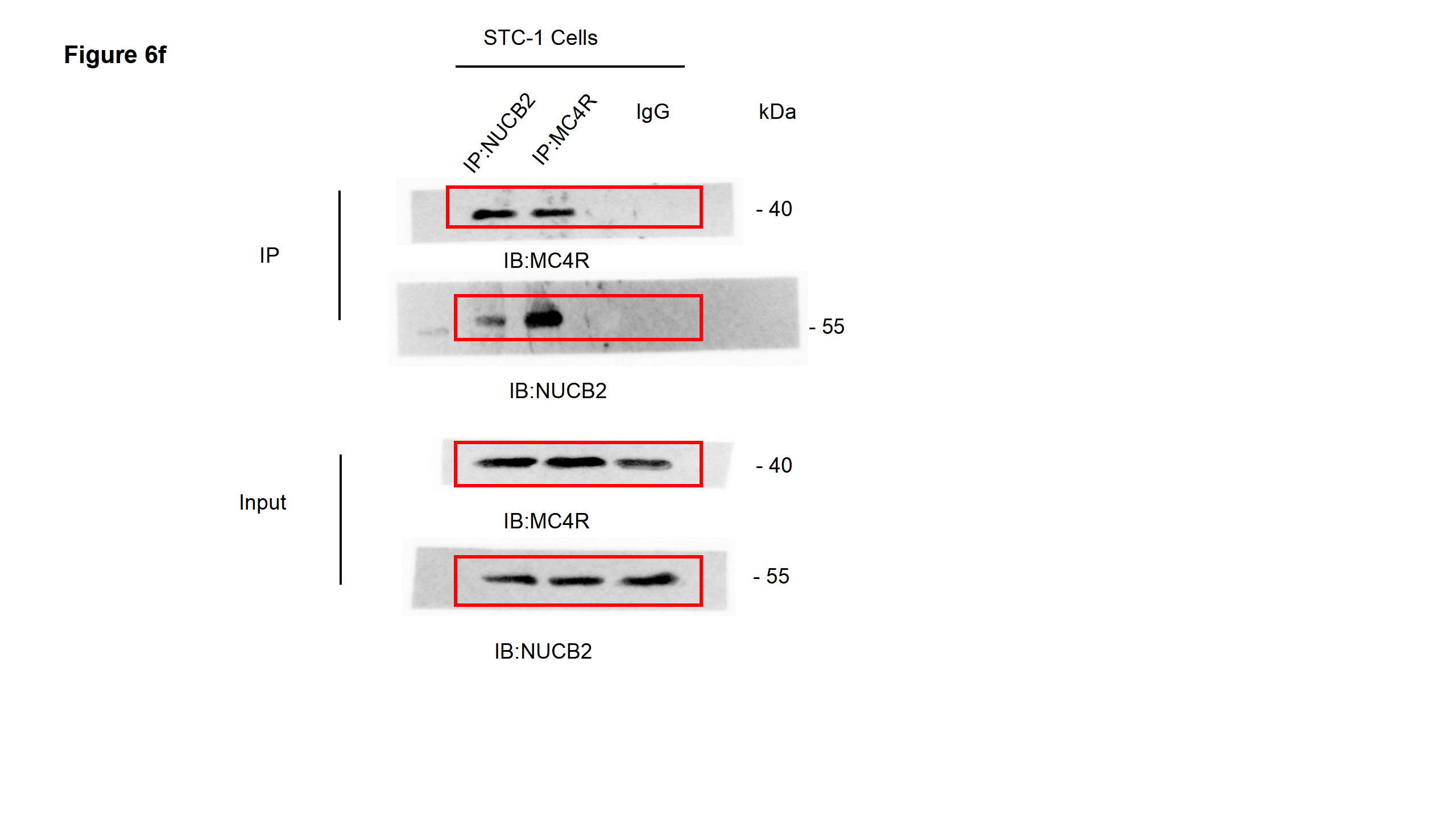

Supplement: Supplementary file 8 — Source data Fig. 6 [file 44318_2024_300_MOESM8_ESM.zip › Figure 6/fig6f/fig6f.tif]

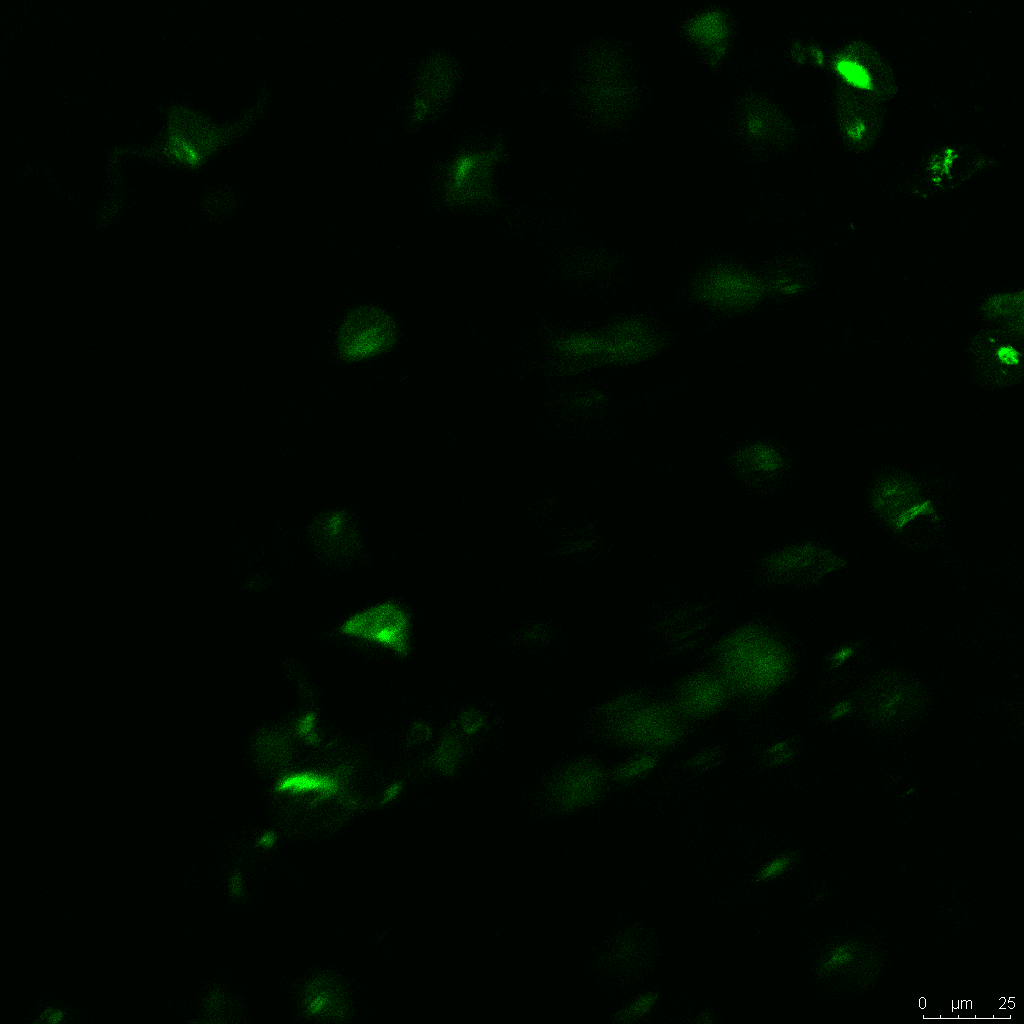

Supplement: Supplementary file 8 — Source data Fig. 6 [file 44318_2024_300_MOESM8_ESM.zip › Figure 6/fig6i/Experment-pGFP-NUCB2-After.tif]

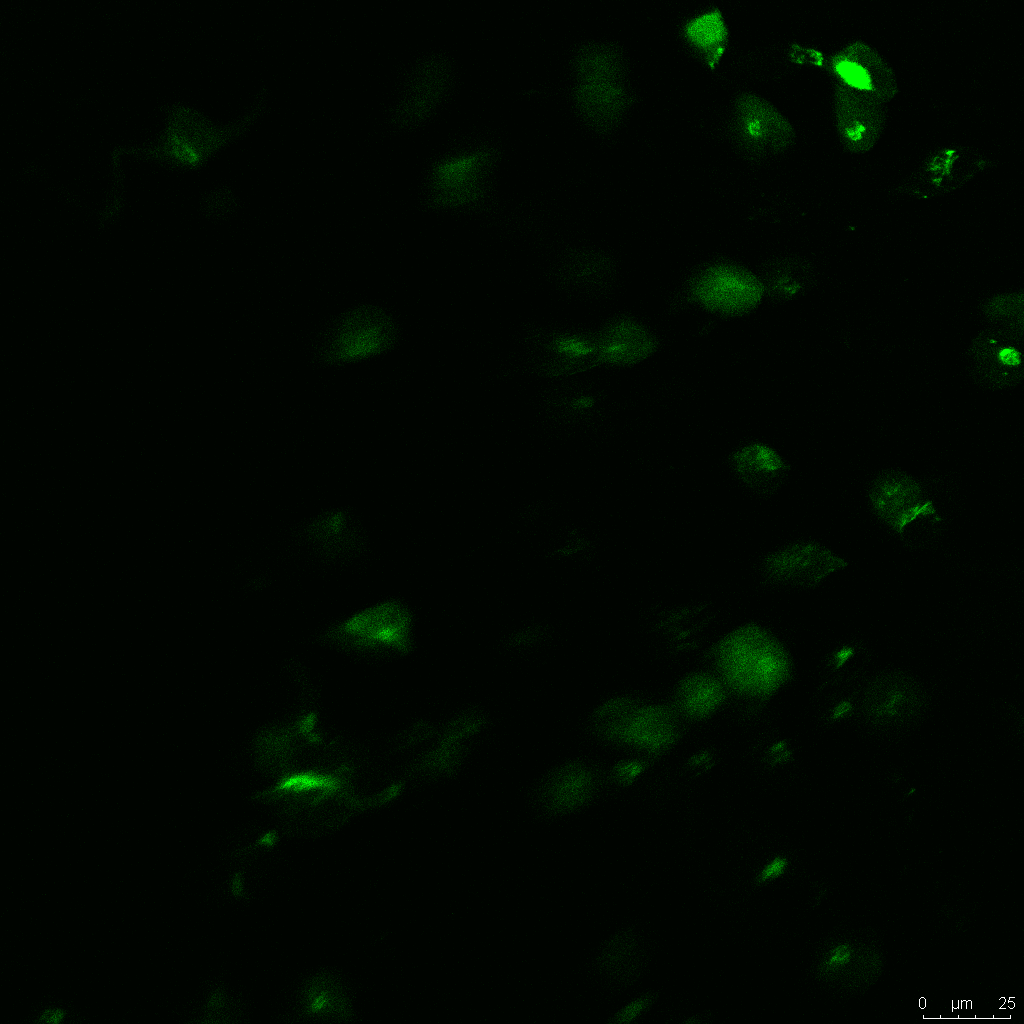

Supplement: Supplementary file 8 — Source data Fig. 6 [file 44318_2024_300_MOESM8_ESM.zip › Figure 6/fig6i/Experment-pGFP-NUCB2-Before.tif]

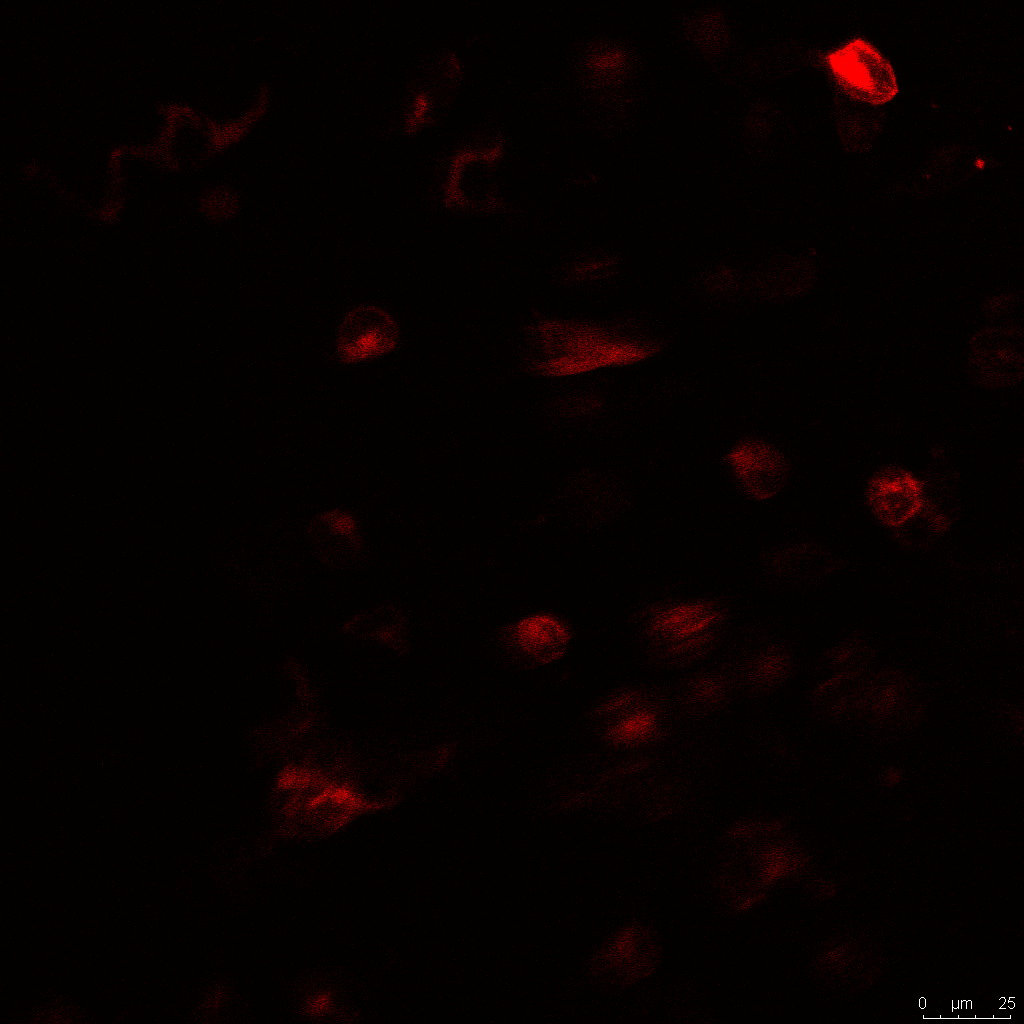

Supplement: Supplementary file 8 — Source data Fig. 6 [file 44318_2024_300_MOESM8_ESM.zip › Figure 6/fig6i/Experment-pmCherry-MC4R-After.tif]

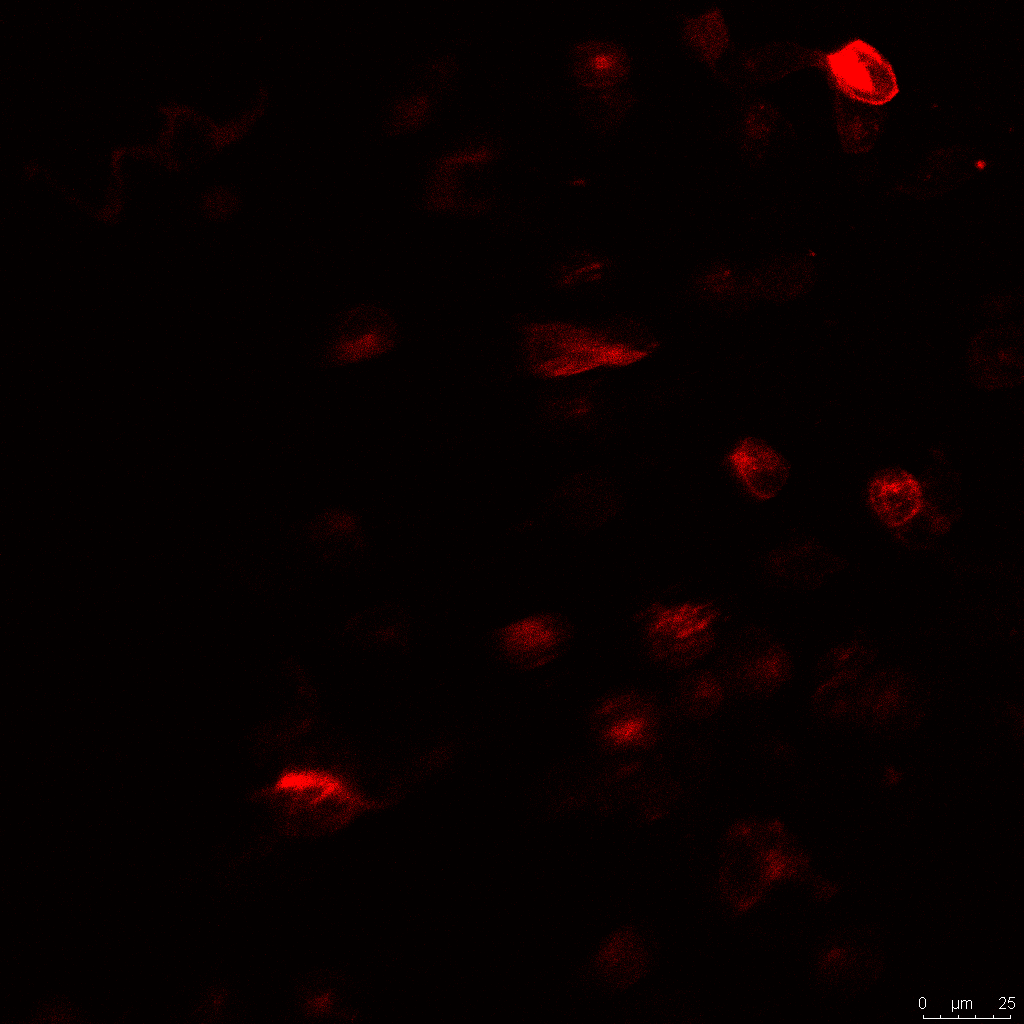

Supplement: Supplementary file 8 — Source data Fig. 6 [file 44318_2024_300_MOESM8_ESM.zip › Figure 6/fig6i/Experment-pmCherry-MC4R-Before.tif]

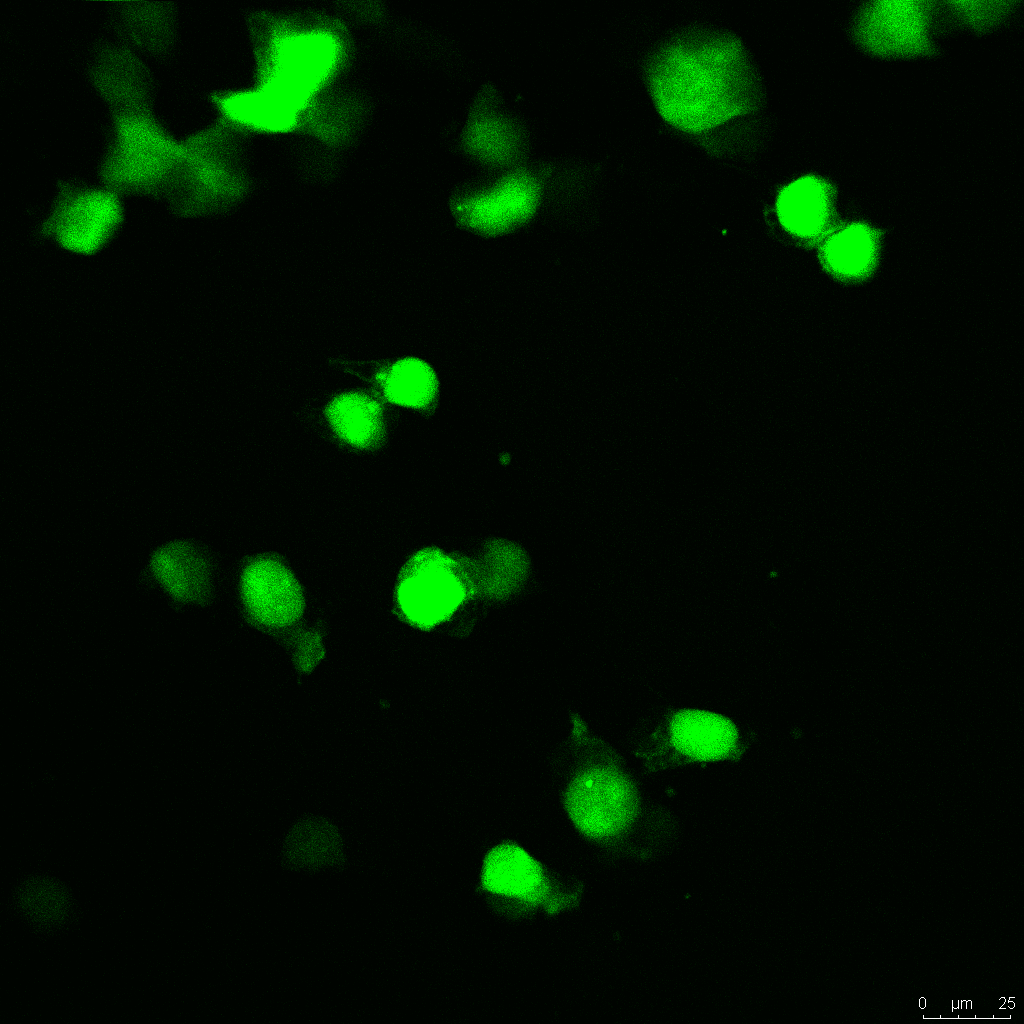

Supplement: Supplementary file 8 — Source data Fig. 6 [file 44318_2024_300_MOESM8_ESM.zip › Figure 6/fig6i/Negaitive-pGFP-NUCB2-After.tif]

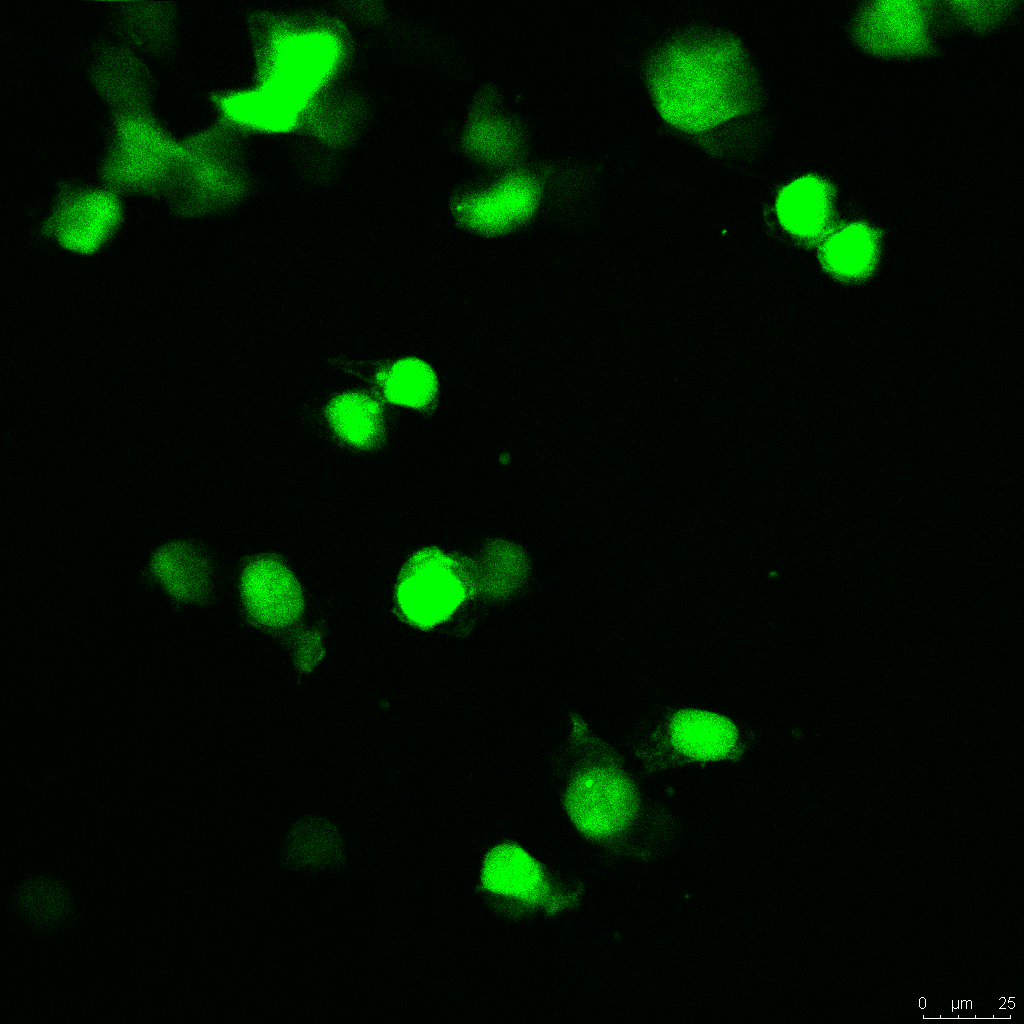

Supplement: Supplementary file 8 — Source data Fig. 6 [file 44318_2024_300_MOESM8_ESM.zip › Figure 6/fig6i/Negaitive-pGFP-NUCB2-Before.tif]

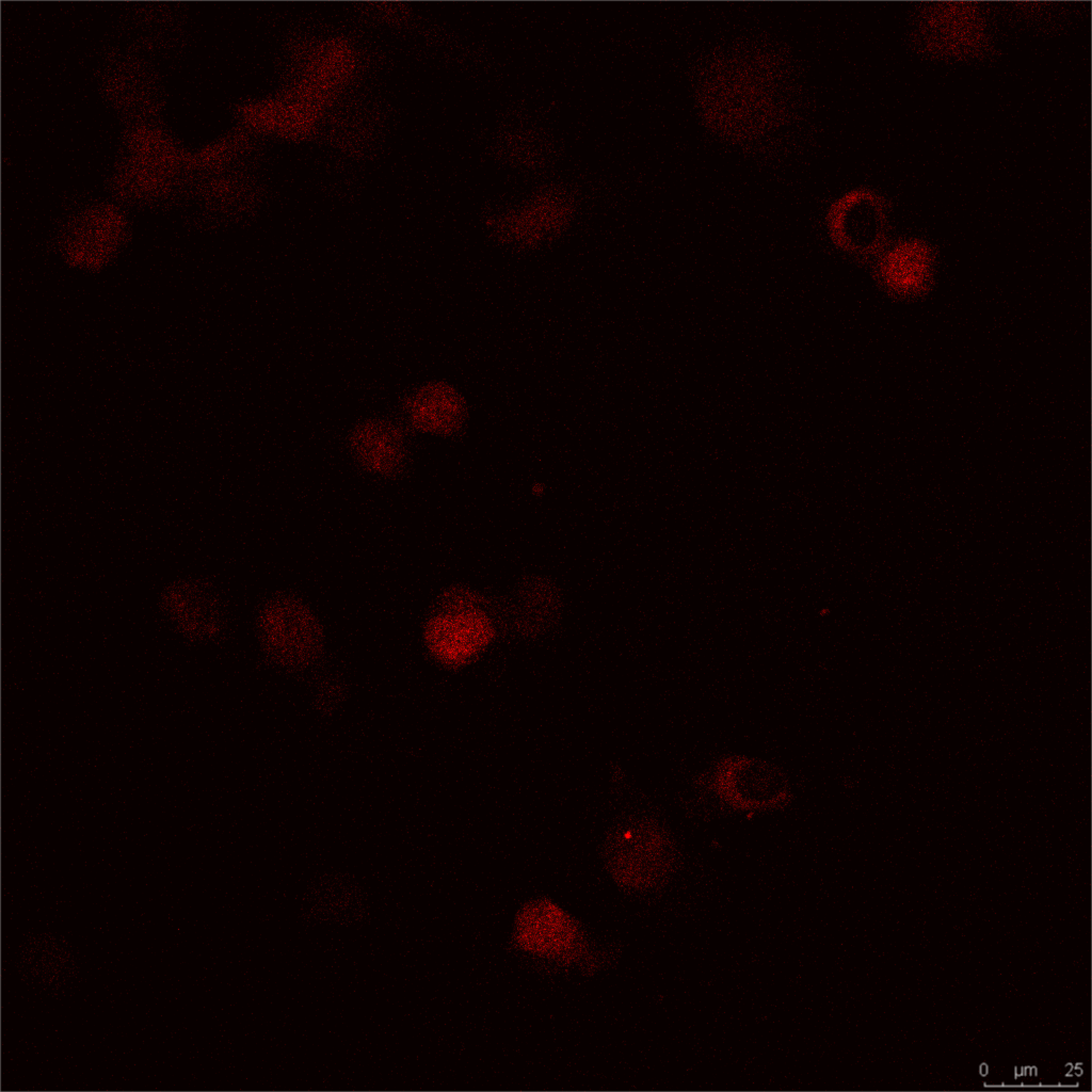

Supplement: Supplementary file 8 — Source data Fig. 6 [file 44318_2024_300_MOESM8_ESM.zip › Figure 6/fig6i/Negaitive-pmCherry-MC4R-After.tif]

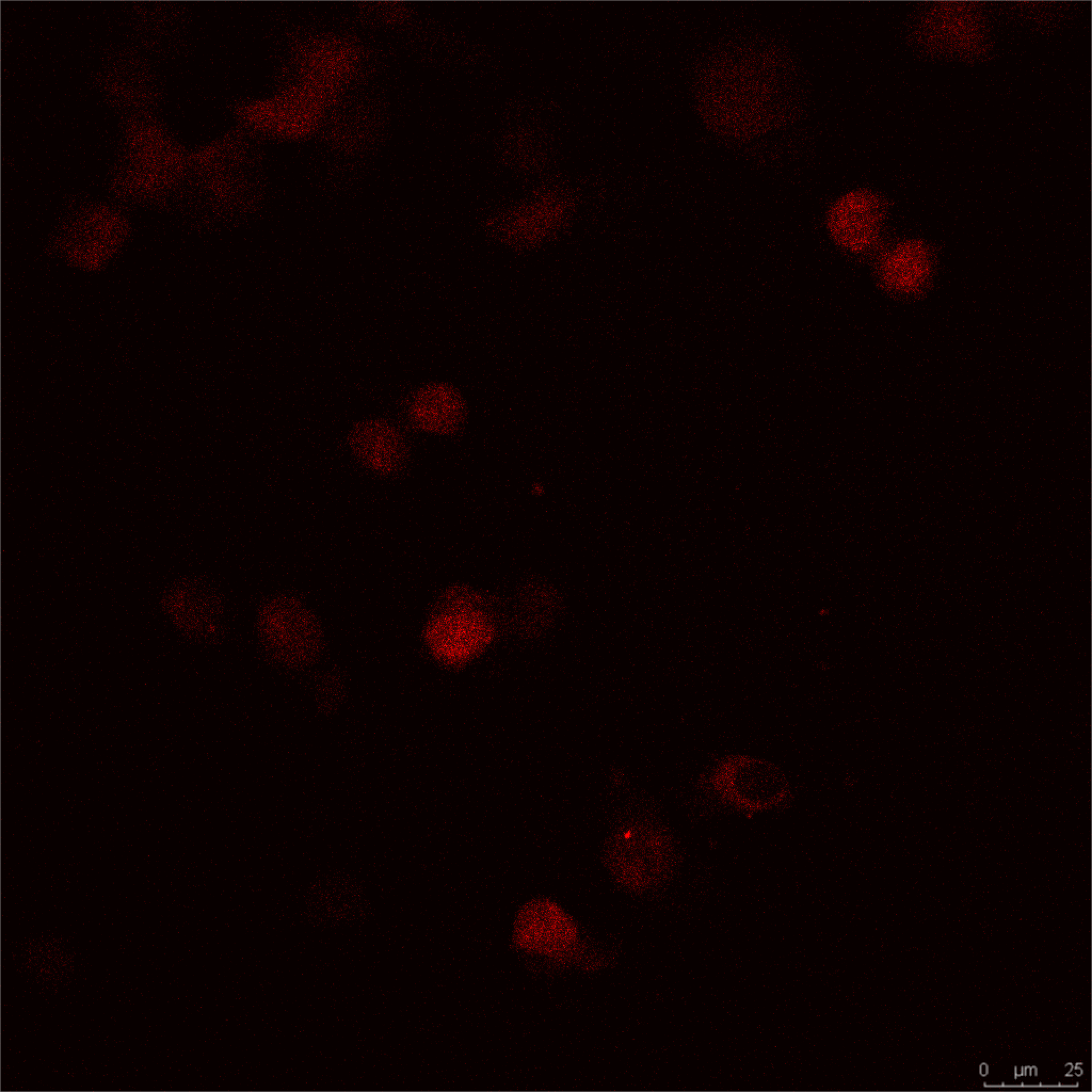

Supplement: Supplementary file 8 — Source data Fig. 6 [file 44318_2024_300_MOESM8_ESM.zip › Figure 6/fig6i/Negaitive-pmCherry-MC4R-Before.tif]

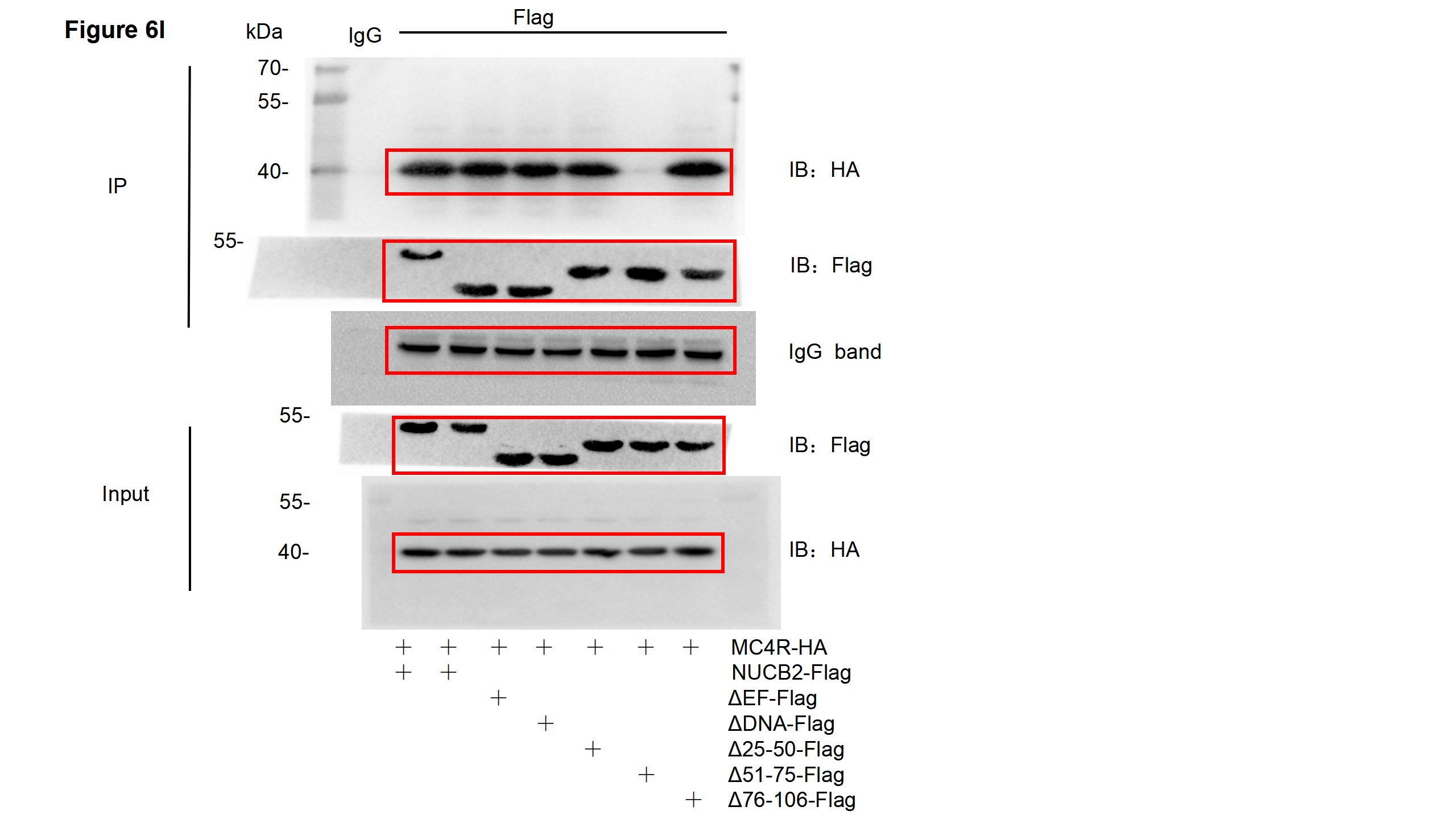

Supplement: Supplementary file 8 — Source data Fig. 6 [file 44318_2024_300_MOESM8_ESM.zip › Figure 6/fig6l/fig6l.tif]

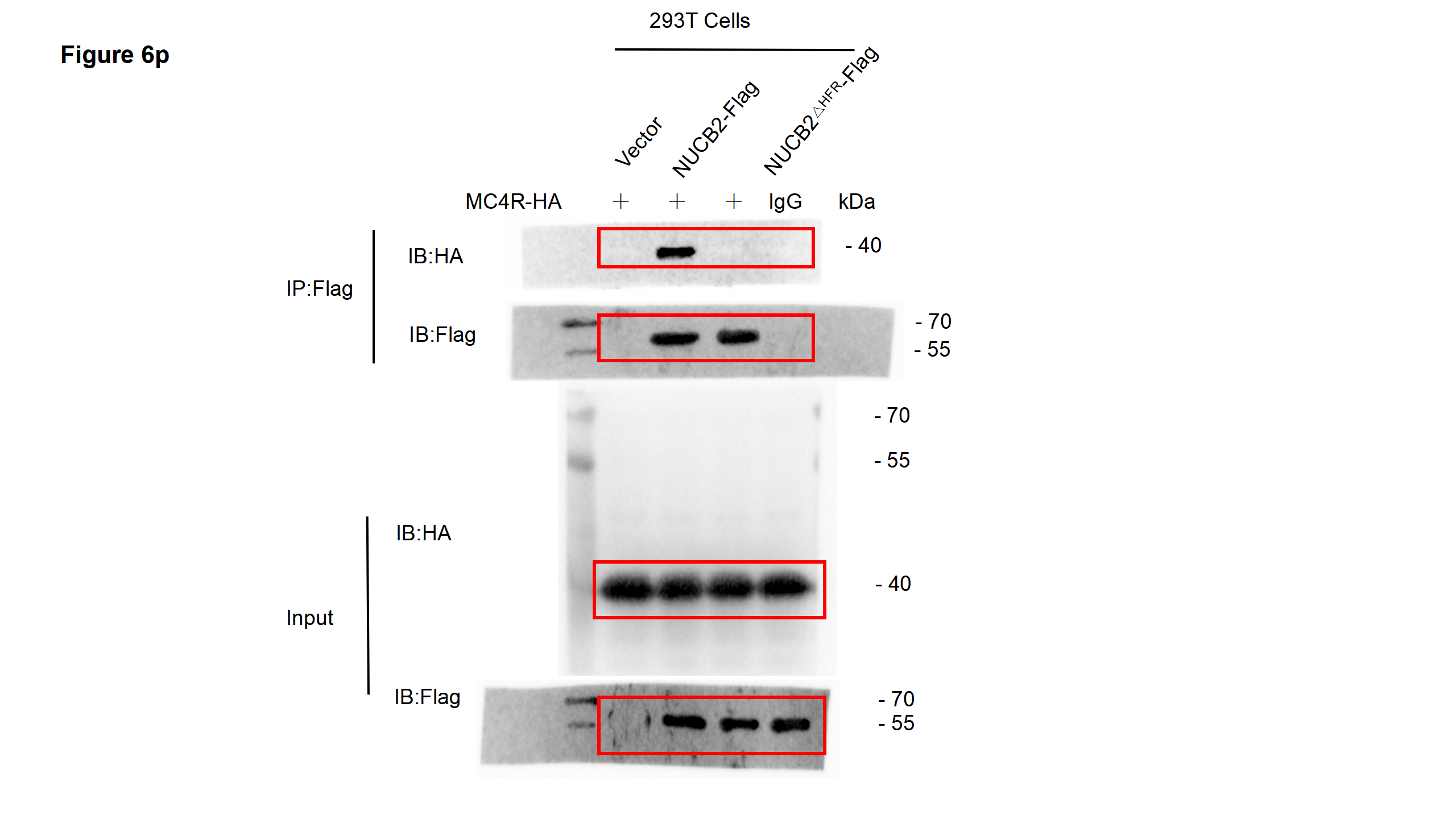

Supplement: Supplementary file 8 — Source data Fig. 6 [file 44318_2024_300_MOESM8_ESM.zip › Figure 6/fig6p/fig6p.tif]
